# Allergens and Other Harmful Substances in Hydroalcoholic Gels: Compliance with Current Regulation

**DOI:** 10.3390/mps6050095

**Published:** 2023-10-07

**Authors:** Ana Castiñeira-Landeira, Lua Vazquez, Thierry Dagnac, Maria Celeiro, María Llompart

**Affiliations:** 1 CRETUS, Department of Analytical Chemistry, Nutrition and Food Science, Universidade de Santiago de Compostela, E-15782 Santiago de Compostela, Spain; anacastineira.landeira@usc.es (A.C.-L.); lua.vazquez.ferreiro@usc.es (L.V.); 2 Galician Agency for Food Quality, Agronomic Research Centre (AGACAL-CIAM), Unit of Organic Contaminants, P.O. Box 10, E-15080 A Coruña, Spain; thierry.dagnac@xunta.gal

**Keywords:** hydroalcoholic gels, personal care products, solid phase microextraction, gas chromatography, tandem mass spectrometry

## Abstract

Hydroalcoholic gels or hand sanitisers have become essential products to prevent and mitigate the transmission of COVID-19. Depending on their use, they can be classified as cosmetics (cleaning the skin) or biocides (with antimicrobial effects). The aim of this work was to determine sixty personal care products frequently found in cosmetic formulations, including fragrance allergens, synthetic musks, preservatives and plasticisers, in hydroalcoholic gels and evaluate their compliance with the current regulation. A simple and fast analytical methodology based on solid-phase microextraction followed by gas chromatography–tandem mass spectrometry (SPME-GC-MS/MS) was validated and applied to 67 real samples. Among the 60 target compounds, 47 of them were found in the analysed hand sanitisers, highlighting the high number of fragrance allergens (up to 23) at concentrations of up to 32,458 μg g^−1^. Most of the samples did not comply with the labelling requirements of the EU Regulation No 1223/2009, and some of them even contained compounds banned in cosmetic products such as plasticisers. Method sustainability was also evaluated using the metric tool AGREEPrep, demonstrating its greenness.

## 1. Introduction

Due to the COVID-19 (severe acute respiratory syndrome coronavirus 2, SARS-CoV-2) infectious disease that was declared a pandemic by the World Health Organization (WHO) in March 2020, promoting hand hygiene by means of alcohol-based hand rub (ABHR) has been considered the primary strategy to mitigate its transmission and infection [1]. In this sense, the production and consumption of hand sanitisers drastically increased worldwide (by up to 561% in Italy, one of the most affected European countries) [2,3]. These products were massively placed in public areas such as shopping centres, schools, banks, supermarkets for public use. Hand sanitisers can be defined as ‘borderline products’ since their classification as a biocide or as a cosmetic product is not clear; this depends on the presence of an active substance and the product’s main purpose [4]. Those designed to disinfect hands, eliminating the microorganisms and their possible transmission, are considered as biocides and are subject to Biocidal Products Regulation (EU) No 528/2012 [5]. On the other hand, if their main purpose is cleaning or cleansing the skin, notably in the absence of water rinsing, then they are considered cosmetics, and probably do not protect through biocidal action. In this case, they must comply with the Cosmetics Regulation (EU) No 1223/2009 [6].

To homogenise their formulation and fabrication, ensuring their antimicrobial properties in the context of the COVID-19 pandemic, the WHO published a protocol, and two formulations were established, containing: (i) ethanol (96%), hydrogen peroxide (3%) and glycerol (98%) and (ii) isopropyl alcohol (99.8%), hydrogen peroxide (3%) and glycerol (98%) [7]. This protocol also strongly recommended that no ingredients other than those specified above be added to the formulation, whilst the addition of fragrances was not recommended because of the risk of allergic reactions. Although it is well known that fragrances are the main responsible of allergic contact dermatitis (ACD), they are usually added to cosmetics with the intention of providing a pleasant scent. Other compounds such as preservatives and plasticisers can also be present in cosmetics and daily care products. Preservatives are added to protect products and consumers against microbial growth, whereas plasticisers (mainly phthalates and adipates) are added to fix and dissolve fragrances, although some can be transferred from the plastic containers to the product and then to consumers, causing health problems, since many of them are catalogued as endocrine disruptors [8,9,10,11].

Different studies have demonstrated that SARS-CoV-2 was efficiently inactivated by WHO-recommended formulations, reducing the viral load to a background level within 30 s, whereas formulations containing other additives present a lower effectivity against the virus [12,13,14].

Therefore, the main objective of this work was the validation and application of a sensitive analytical method to simultaneously determine fragrances (allergens and synthetic musks) and other potentially harmful substances, such as plasticisers and preservatives, including 60 compounds, in a broad range of hand sanitiser samples. In general, the analysis of cosmetics and personal care products is a challenge for analysts, as ingredients may be present at % concentrations, or at trace levels in the case of impurities. A suitable option for the multianalyte analysis of these matrices is the use of solid-phase microextraction (SPME), a simple, fast and environmentally friendly extraction technique, followed by gas chromatography–tandem mass spectrometry (GC-MS/MS). This combination has been reported as a reliable and sensitive tool to analyse a high number of personal care products (PCPs) in different cosmetic products, including hydroalcoholic gels [15,16,17,18]. The method was employed to conduct a survey of hand sanitisers for personal use (personal use) and placed in local shops or public areas (public use) to verify compliance with applicable legislation. A labelling study was also carried out to check whether the information for consumers was correct and complete. To the best of our knowledge, there are no analytical studies dedicated to researching this type of samples used daily by a large part of the world’s population since the COVID-19 pandemic.

## 2. Materials and Methods

### 2.1. Chemicals, Reagents and Materials

The 60 target compounds, their CAS numbers, molecular mass, European legislation restrictions, retention times and MS/MS transitions are shown in Table S1.

Ultrapure water and methanol, both MS grade, were purchased from Scharlau (Barcelona, Spain) and acetone from Sigma Aldrich Chemie GmbH (Steinheim, Germany). Moreover, 50/30 μm of commercial divinylbenzene/carboxen/polydimethylsiloxane (DVB/CAR/PDMS) fibre housed in a manual SPME holder was obtained from Supelco (Bellefonte, PA, USA). Prior to the first use, the fibre was conditioned as recommended by the manufacturer by inserting it into the GC injector with carrier gas flow at 270 °C for 30 min.

The target compounds were selected, including 23 fragrance allergens, 11 synthetic musks, 16 plasticisers and 10 preservatives. Individual stock solutions were prepared in methanol or ethyl acetate, followed by further dilutions in acetone. Working solutions were prepared weekly. All of them were stored in amber glass vials and protected from light at −20 °C.

### 2.2. Samples and Sampling

Sixty-seven hydroalcoholic gel samples were collected from different establishments in Galicia (Northwest Spain), including banks, restaurants, pharmacies, universities, local markets in 15 mL amber vials. The samples were kept at room temperature and protected from light until analysis. Sampling details about the establishments where they were collected, and the composition indicated in the label, are summarised in Table S2.

### 2.3. Sample Preparation Procedure: SPME

Herein, 10 mg of hydroalcoholic gel and 10 mL of ultrapure water (1:1000, *w*/*v* dilution) were placed in a 20 mL glass vial, which was sealed with an aluminium cap furnished with PTFE-faced septa. The vial with the sample was immersed in a water bath at 100 °C. First, the sample was conditioned during 5 min and then the DVB/CAR/PDMS fibre was exposed for 20 min to the headspace over the sample (HS-SPME mode). Agitation under magnetic stirring was employed during the extraction procedure. After the extraction time, the SPME fibre was desorbed at 270 °C for 5 min in the GC injection port and the GC-MS/MS analysis was carried out.

In some cases, the high concentration of some of the analytes in the samples made it necessary to perform a further dilution of the hydroalcoholic gel–water mixture in ultrapure water, followed by the corresponding SPME GC-MS/MS analysis.

Since one of the studied families are plasticisers, and to avoid contamination and overestimation in the results, the plastic material was replaced with glass and metallic. All material was also maintained at 230 °C for 12 h before use. In addition, fibre blanks and procedure blanks using 10 mL of ultrapure water were carried out with the aim of avoiding false-positive results.

### 2.4. GC-MS/MS Analysis

The GC-MS/MS analysis was performed employing a Thermo Scientific Trace 1310 gas chromatograph coupled to a triple-quadrupole mass spectrometer (TSQ 8000) from Thermo Scientific (San Jose, CA, USA).

Separation was performed on a Zebron ZB-Semivolatiles (30 m × 0.25 mm i.d. × 0.25 µm film thickness) obtained from Phenomenex (Torrance, CA, USA). Helium (purity 99.999%) was used as carrier gas at a constant column flow of 1 mL min^−1^. The GC oven temperature was programmed from 60 °C (held 1 min), to 100 °C at 8 °C min^−1^, to 150 °C at 20 °C min^−1^, to 200 °C at 25 °C min^−1^ (held 5 min), to 220 °C at 8 °C min^−1^, and finally, to 290 °C at 30 °C min^−1^ (held 7 min). The total run time was 30 min. Split/splitless mode was used for injection (200 kPa, held 1.2 min) and the injector temperature was kept at 270 °C.

The mass spectra detector (MSD) was operated in the electron impact (EI) ionisation positive mode (+70 eV). The temperatures of the transfer line and the ion source were established at 290 and 350 °C, respectively. The filament was set at 25 µA and the electron multiplier was set at a nominal value of 1800 V. Two or three transitions were monitored per compound working in the selected reaction monitoring (SRM) acquisition mode, as can be seen in Table S1. The system was operated by Xcalibur 2.2, and Trace Finder^TM^ 3.2 software.

## 3. Results and Discussion

### 3.1. SPME-GC-MS/MS—Method Validation

The SPME experimental parameters, including fibre coating, extraction mode, temperature and dilution factor were previously optimised [17], and the most suitable conditions are described in Section 2.

The miniaturised method, employing only 10 mg of sample, was validated in terms of linearity, repeatability, accuracy and reproducibility. Instrumental detection limits (IDLs) and limits of detection (LODs) were also calculated. The results are summarised in Table S3. External calibration was assessed by preparing the standards in ultrapure water and following the procedure detailed in Section 2.3 covering a concentration range between 0.02 and 5 μg L^−1^ (see specific ranges for each target analyte in Table S3). The method showed coefficients of determination (R^2^) higher than 0.9901, demonstrating a direct proportional relationship between the amount of the target compound with the corresponding chromatographic response (area counts).

Precision was evaluated within a day (*n* = 4) and among several days (*n* = 6) for all the calibration levels. Table S3 shows relative standard deviation (RSD) values for 1 µg L^−1^. As can be seen, they were lower than 13% and 16% for repeatability and reproducibility, respectively, in most cases. Accuracy was verified using an hydroalcoholic gel sample free of the target compounds which was fortified at two levels (0.2 µg g^−1^ and 2 µg g^−1^). Mean recoveries between 76 and 117% and precision (RSD%) < 10% were obtained, as can be seen in Figure S1.

IDLs were calculated as the compound concentration giving a signal-to-noise ratio of three (S/N = 3), employing ultrapure water standards containing low concentrations of the target analytes. LODs were calculated employing a real sample spiked with the target compounds. For the plasticisers that were detected in the procedure blanks (DEP, DBP, DEHP, DPhP, and DEHA), IDLs and LODs were calculated as the mean concentration corresponding to the signal of the blanks plus three times its standard deviation. Results are summarised in Table S3 and they were at the low ng g^−1^ level for all compounds. In this case, it should be kept in mind that the gel samples are diluted by a factor of 1/1000, which explains the difference between IDL and LOD values. If higher sensitivity were required, a low dilution factor could be applied.

### 3.2. Greenness Assessment

In recent years, the development of extraction methodologies fulfilling green analytical chemistry (GAC) and green sample preparation (GSP) principles is increasing. These procedures include the use of safe solvents, reagents and materials minimising the experimental steps and reducing waste generation and energy consumption, allowing high sample throughput. In 2022, a metric tool, AGREEprep [19] was proposed for assessing the greenness of the sample preparation stage of an analytical procedure. The metric is based on ten principles of GSP, assigning weights at each criteria, depending on its significance in the analytical method. These weights range from 1, indicating low importance, to 5, representing high importance. Once the ten principles are evaluated, the tool is recalculated to the 0–1 scale to reflect the sample preparation greenness. The colour of the inner circle also provides the overall sample preparation greenness performance.

**Figure 1:**
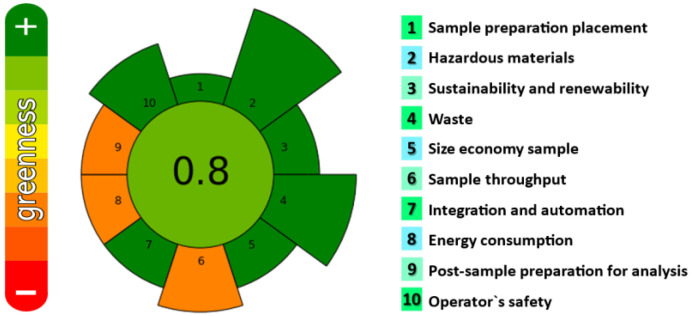
Evaluation of the degree of greenness. Pictogram obtained for SPME-GC-MS/MS under optimised conditions.

The sustainability of the SPME-GC-MS/MS method for extracting 60 personal care products from hydroalcoholic gels was calculated and results are depicted in Figure 1.

As can be seen, a value of 0.80 as well as a green label were obtained showing the greenness of the proposed SPME method. Considering each criteria, the developed method procedure is in-line/in situ since the SPME technique enables the integration of sample preparation and analysis (criteria 1); no toxic materials are used (criteria 2); the employed SPME fibre can be used several times (criteria 3); no waste is generated because the samples are hydroalcoholic gels (criteria 4); the sample amount is 0.01 g (criteria 5); the duration of the sample preparation stage is around 20 min; three samples per hour (criteria 6); the procedure consists of two steps, extraction and desorption, and it is a fully automated system (criteria 7); the energy consumption is more than 183 Wh due to the heating magnetic stirrer (criteria 8); GC-MS/MS is used as analytical instrument (criteria 9); and, finally, no hazards are associated with the procedure (criteria 10). The weights of each criteria were, in general, not modified (default weights given by AGREEprep).

### 3.3. Analysis of Real Samples

**Figure 2:**
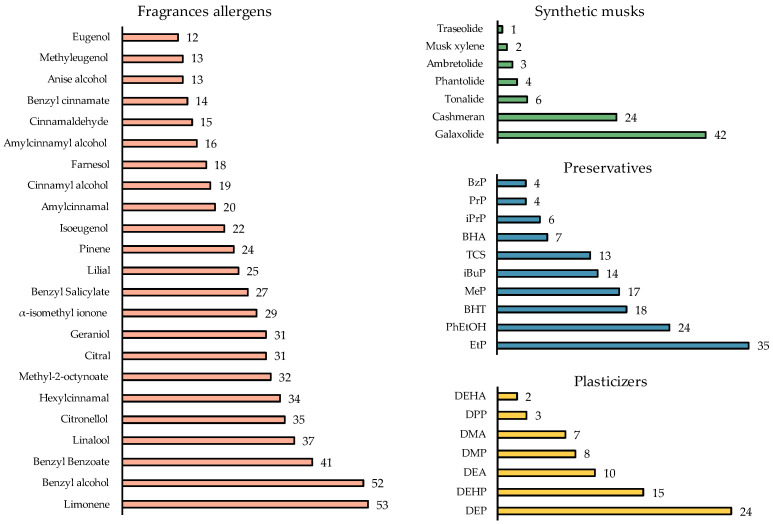
Frequency of the target compounds by families in the hydroalcoholic gels. Numbers indicate how many hydroalcoholic gels samples out of the 67 analysed contain each compound.

The SPME-GC-MS/MS method was applied to analyse the target compounds in 67 hydroalcoholic gels, collected in different public places (see Section 2.2), in which 47 of the 60 target compounds were found (see Figure 2).

**Figure 3:**
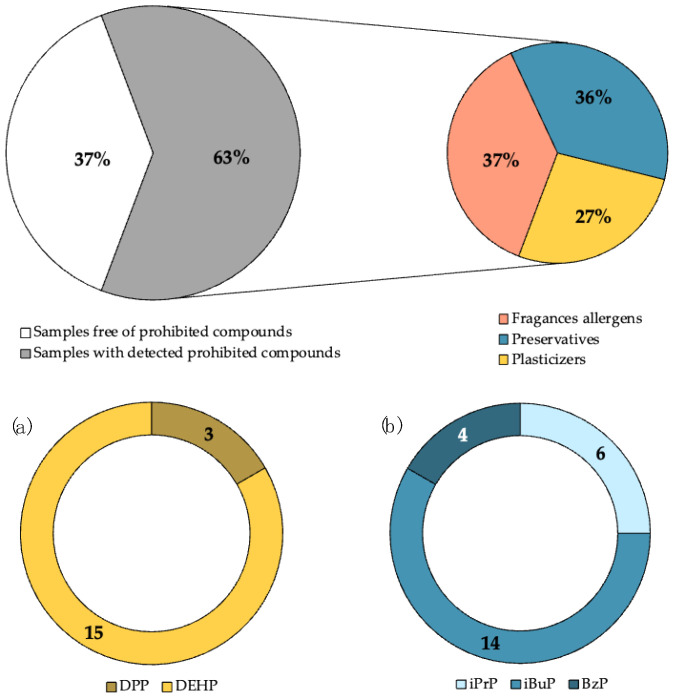
Percentage of banned compounds in the analysed samples and distribution by families: (**a**) phthalates and (**b**) parabens.

Most of the analysed samples do not comply with the WHO recommendations due to the presence of the target compounds (fragrances allergens, synthetic musks, plasticisers and preservatives) in the formulations. In this context, if the sample should be considered a cosmetic product, it must comply the European Cosmetics Regulation (EC) No 1223/2009 [6] in order to ensure product safety. This study highlights that 61% of the hydroalcoholic gels contain at least one compound which is prohibited by the Cosmetic Products Regulation, as can be seen in Figure 3.

A description of the compliance with this regulation is presented below for each studied family. Table 1 summarises the concentration range, mean and median of the target compounds in the analysed samples, and the specific quantification results for the studied compounds in each sample are summarised in Table 2.

#### 3.3.1. Fragrance Allergens

As can be seen in Table 1 and Table 2, all studied fragrance allergens were detected in the real analysed samples. Limonene, benzyl alcohol and benzyl benzoate were found in most samples in 53, 52 and 41 out of the 67 hand sanitisers, respectively. Other fragrances such as linalool (37/67), citronellol (35/67), hexyl cinnamal (34/67), methyl-2-octynoate (32/67), citral and geraniol (31/67) were found in 50% of the samples. The presence of this family of cosmetic ingredients is remarkable since even the least frequently detected fragrances such as eugenol, anise alcohol and methyl eugenol, were found in 20% of the samples (see Figure 2).

Regarding the number of compounds per sample, 7 of the analysed samples contained around 17–19 of the 23 target fragrance allergens (G36, G43, G50, G51, G53, G65 and G66). In contrast, only two were free of these substances, samples G12 and G47, and only one sample (G16) contained one fragrance allergen (citronellol).

It should be underlined that the high levels found for some allergens in some samples reach concentrations of parts per hundred (up to 3%) as amyl cinnamal (32,458 μg g^−1^ in sample G51) and farnesol (28,737 μg g^−1^ in sample G24).

##### Regulatory Issues

According to EC Cosmetic Regulation No. 1223/2009, fragrance allergens can cause allergic skin reactions and other adverse effects especially at high concentrations. Among the 23 studied fragrance allergens, 20 (limonene, benzyl alcohol, linalool, methyl-2-octynoate, citronellol, citral, geraniol, cinnamaldehyde, anise alcohol, cinnamyl alcohol, eugenol, isoeugenol, α-isomethylionone, amyl cinnamal, amylcinnamyl alcohol, farnesol, hexyl cinnamal, benzyl salicylate and benzyl cinnamate) should appear on the label of cosmetic products when the concentration exceeds 0.001% (*w*/*w*, 10 μg g^−1^) in leave-on products, as is the case for hand sanitisers [6]. Among the 67 analysed samples, 33 (49%) exceed this limit for several compounds; in addition, most of they are under-labelled since the corresponding fragrances are not included in the product label. In contrast, some hydroalcoholic gels were over-labelled, as they claimed to contain more allergens than they did (over-labelling is not included in the regulation). For example, sample G24 indicates the presence of benzyl benzoate, which is over-labelled since it does not surpass its legal limit (0.001%, 10 μg g^−1^). In addition, two out of the studied fragrances present a maximum permitted concentration in final products as methyl-2-octynoate with 0.01% and isoeugenol with 0.02%. The sample G36 is the only one which surpassed the isoeugenol limit with a concentration of 739 μg g^−1^.

Some fragrance allergens are prohibited, such as lilial, which has been banned since March 2022 because it damages fertility and it is suspected of damaging the unborn child, in addition to causing skin irritation [20]. Lilial was present in around the 37% (Figure 2) of the analysed hydroalcoholic gels (25/67) at concentrations below 0.001% (*w*/*w*) (10 μg g^−1^) in 22 samples. Three samples (G10, G24 and G29) contained between 7 and 15 μg g^−1^ of lilial, highlighting one sample which presented a very high value (517 μg g^−1^, G20). However, it should be pointed out that some hydroalcoholic gel samples were taken after the lilial legislation had changed.

#### 3.3.2. Synthetic Musks

Regarding the synthetic musks, as can be seen in Table 3, 7 of the 11 targets were found in the analysed hydroalcoholic gels, highlighting the presence of galaxolide in 63% of the samples (42/67), followed by cashmeran in 36% (24/67).

Only 20% of the samples were free of these compounds (16/67). The highest number of synthetic musks (galaxolide, cashmeran, phantolide and tonalide) were detected in sample G33. Although several of these PCPs such as galaxolide were found at concentrations above 10 μg g^−1^ (e.g., 56 μg g^−1^ in G33), in most cases, these compounds were presented at concentrations below 1 μg g^−1^. An exception is the musk xylene which was detected in two samples at extraordinarily high concentrations, 2356 μg g^−1^ in G65 and 1840 μg g^−1^ in G66; this being the synthetic musk with the highest concentrations followed by cashmeran at 74 μg g^−1^ in G67.

##### Regulatory Issues

The regulation of cosmetic products states that the synthetic musks ambrette, tibetene and moskene are prohibited. These chemicals were not detected in any of the analysed hydroalcoholic gels. Nevertheless, 51 samples contained at least one target synthetic musk, so the terms “parfum” or “aroma” must appear on their label. However, only 17 samples (see Table S2) were labelled with the word ‘parfum’, which indicates that 34 of 51 are under-labelled, although in general, the concentrations for this family of fragrances were quite low as it was commented. Some substances such as tonalide, phantolide and musk xylene must be mentioned when their concentration surpass 1%, 2% and 0.03%, respectively. Musk xylene was detected in two samples at very high concentrations, surpassing the regulation’s limit, and its presence was not indicated in the label of any sample. In the case of phantolide and tonalide, they were detected in four and six samples, respectively, but none of them surpassed the regulation’s limit, so they did not have to be indicated, as it is the case. As was mentioned, galaxolide was the most frequently found (63% of the analysed samples) and is under assessment as a persistent, bioaccumulative and endocrine disruptor [21].

#### 3.3.3. Preservatives

All target preservatives were found in the analysed samples, as can be seen in Table 4.

The most frequently found were EtP in half of the samples (52%) and PhEtOH in 37% (25/67). The antioxidant BHT and the parabens MeP and iBuP were found in more than 20% of the samples and TCS and iBuP were detected in more than 10 samples.

The highest number of preservatives found in the same sample was six in G29, G51 and G53. Nevertheless, more than the 56% of the samples contained between 1 (25/67) and 2 (13/67) of the target preservatives. In six samples, none of the target preservatives were present (G14, G26, G40, G47, G60 and G64).

In most cases, the concentrations were at the low μg g^−1^ (Table 4), excluding PhEtOH which was found at very high concentrations of up to 13,735 μg g^−1^. The median concentration value of this substance in the analysed samples was 20 μg g^−1^, which is the highest value with a noticeable difference with the rest of the preservatives. Regarding the detected parabens, PrP was presented at concentrations of up to 150 μg g^−1^, EtP, iBuP and BzP up to 60 μg g^−1^ and MeP up to 23 μg g^−1^. BHT was detected at concentrations of up to 170 μg g^−1^ and TCS and BHA only appear at very low concentrations below 1 μg g^−1^.

##### Regulatory Issues

The Annex V of the EC Regulation No 1223/2009 comprised the preservatives allowed in cosmetic products, among which some of the target substances are included (PhEtOH, MeP, EtP and PrP).

The established maximum concentration in ready-for-use preparation for PhEtOH, a substance which is harmful if ingested, causing serious eye damage and may cause respiratory irritation, is 1.0% (10,000 μg g^−1^). However, samples G49 and G22 surpassed this limit since they contained 1.4 and 1.0% (*w*/*w*) of the sample, respectively. The labels of these hydroalcoholic gels in addition to G35, G37 and G44 show that this compound is present at 2.1% (*w*/*w*). As described in Table S2, these samples comply with UNE-EN standards and, although all samples have the same labelling, the brand name is different, so they were analysed individually. The appearance of this compound in the product labelling in such high concentrations is due to these hydroalcoholic gels being considered biocides and that ECHA has endorsed the approval of PhEtOH as an active substance in type 1 biocidal product, which are those intended for human hygiene [5,22]. Nevertheless, they contained other substances such as fragrances, so they should be considered cosme-tics, although some of them would not comply with this regulation.

As regard the parabens EtP and MeP, PrP were detected at concentrations lower than the permitted limits, 0.4% (EtP and MeP) and 0.14% (PrP). The prohibited parabens in cosmetic products, as iPrP, iBuP and BzP, were found in around 9%, 21% and 6%, respectively, of the analysed hand sanitisers at concentrations of up to 60 μg g^−1^ (iBuP in G4 and BzP in G56).

The antioxidant BHT, which has been very recently (July 2023) included in the Annex III of Regulation EC 1223/2009—substances in cosmetics products must not contain except subject to the restrictions laid down—is regulated for leave-on and rinse-off products at a maximum concentration of 8000 μg g^−1^, but none of the hydroalcoholic gels surpassed this value since the highest concentration was 170 μg g^−1^ in G24. In contrast, the triclosan is regulated but it does not have a limit for this type of cosmetic products.

#### 3.3.4. Plasticisers

Considering plasticisers, four phthalates and three adipates were present in the analysed hydroalcoholic gels: DMP (8/67), DEP (24/67), DPP (3/67), DEHP (15/67) and DMA (7/67), DEA (10/67), DEHA (2/67), as can be seen in Table 5.

Regarding the number of compounds, in most samples, two or three of the target compounds were found. One sample presented five of the compounds (G51) followed by the sample G39 with four compounds (Table 5). Almost half of the samples (31) were free of the target plasticisers.

In general, the concentrations were below 2 μg g^−1^ but it should be underlined that there is the high concentration of DEP in some samples with concentrations between 1800 and 8240 μg g^−1^ (0.2–1%). DMP reached a concentration of 108 μg g^−1^ in sample G28, although this value is much lower than the previous values indicated for DEP.

##### Regulatory Issues

Some of the analysed plasticisers are banned by the EC Regulation No 1223/2009. Among the detected ones, DEHP and DPP are prohibited in cosmetics (see Figure 3), and they were detected in 3 and 15 samples, respectively. Both substances are toxic for reproduction and DEHP is also considered an endocrine disruptor. The presence of these phthalates may be due to migration from the plastic container to the hydroalcoholic gel. Previous studies have demonstrated the transfer of certain substances from the applicators to cosmetic products [23]. Other prohibited substances such as DBP, DMEP, DIPP, BBP, DIBP, DIHP, DPhP, DnOP and DCHP were not found in any of the analysed samples. On the other hand, it is important to note that DEP, the plasticiser found in the highest concentrations (up to 8238 μg g^−1^), is under evaluation as an endocrine disruptor, even though it is not legislated in cosmetic products.

## 4. Conclusions

Sixty-seven hydroalcoholic gels collected in different establishments were analysed by SPME-GC-MS/MS, a validated methodology which allows a complete analysis of the hydroalcoholic gel samples. Sixty personal care products, including fragrance allergens, synthetic musks, preservatives and plasticisers were targeted. The results revealed the presence of 48 out of these 60 target compounds in the analysed hydroalcoholic gels. Only one sample complied with the WHO recommendations for hand sanitiser formulations. The highest concentrations were observed for fragrance allergens and many of the samples did not comply with the legislation for these substances as, despite not being labelled, they contained some above the 0.001% limit in leave-on products. Furthermore, some prohibited compounds (iPrP, iBuP, BzP, DEHP and DPP) were detected in some cases. In addition, some hydroalcoholic gels are considered biocidal, although after the analysis of their composition, they should be considered cosmetic products. In this context, PhEtOH sometimes exceeds the limits set for cosmetics, while it is allowed for biocides for human hygienic use. Therefore, results demonstrated that greater control over the formulations of these frequently used cosmetic products is necessary to ensure consumer safety without causing undesirable side effects.

## Figures and Tables

**Table 1:** Number of detected compounds, concentration range, mean and median, expressed at μg g^−1^ in 67 analysed samples.

	Number	Range	Mean	Median
**Fragrance Allergens**	**65**	**0.0054–32,458**	**180**	**0.801**
Pinene	24	0.022–4.4	1.0	0.59
Limonene	53	0.057–177	20	0.77
Benzyl alcohol	52	0.044–212	22	1.3
Linalool	37	0.036–209	35	4.9
Methyl-2-octynoate	32	0.072–72	7.1	1.25
Citronellol	35	0.0085–53	8.8	2.0
Citral	31	0.20–102	15	5.1
Geraniol	31	0.070–1747	103	12
Cinnamaldehyde	15	0.14–59	5.3	0.31
Anise alcohol	13	0.076–17,035	1375	11.7
Cinnamyl alcohol	19	0.17–327	51	3.7
Eugenol	12	0.026–110	23	6.5
Methyl eugenol	13	0.011–4.7	0.44	0.083
Isoeugenol	22	0.031–739	57	2.1
α-isomethyl ionone	29	0.0054–130	7.9	0.12
Lilial	25	0.0063–517	23	0.056
Amyl cinnamal	20	0.0094–32,458	1832	0.11
Amylcinnamyl alcohol	16	0.0072–427	45	0.58
Farnesol	18	0.0613–28,737	2363	7.07
Hexyl cinnamal	34	0.015–755	28	0.19
Benzyl benzoate	41	0.052–259	11	0.29
Benzyl salicylate	27	0.035–154	11	0.14
Benzyl cinnamate	14	0.019–0.84	0.15	0.058
**Synthetic musks**	**51**	**0.0042–2356**	**56**	**0.16**
Musk xylene	2	1840–2356	2098	2098
Galaxolide	42	0.019–56	5.8	0.17
Phantolide	4	0.011–29	7.3	0.081
Cashmeran	24	0.0042–74	4.2	0.16
Traseolide	1	1.8	1.8	1.8
Tonalide	6	0.022–0.386	0.131	0.0918
Ambretolide	3	0.0057–4.0	1.4	0.34
**Preservatives**	**61**	**0.0035–13,735**	**365**	**1.59**
PhEtOH	24	0.2121–13,735	2115	20.49
BHA	7	0.012–0.57	0.23	0.13
BHT	18	0.0035–170	10	0.043
TCS	13	0.039–0.45	0.15	0.10
MeP	17	0.18–23	4.4	1.3
EtP	35	0.58–62	9.0	3.6
iPrP	6	0.070–1.1	0.41	0.26
PrP	4	0.15–150	39	2.1
iBuP	14	0.15–61	14	4.1
BzP	4	3.0–59	21	11
**Plasticisers**	**36**	**0.01–8238**	**256**	**0.54**
DMP	8	0.16–108	15	0.56
DEP	24	0.19–8238	731	3.9
DPP	3	0.050–0.14	0.10	0.11
DEHP	15	0.11–12	1.4	0.25
DMA	7	0.11–4.07	0.87	0.23
DEA	10	0.010–1.8	0.48	0.11
DEHA	2	0.40–0.92	0.66	0.66

**Table 2:** Individual concentration (μg g^−1^) of the fragrance allergens detected in 67 hydroalcoholic gel samples and total content.

	Pinene	Limonene	Benzyl Alcohol	Linalool	Methyl-2-Octynoate	Citronellol	Citral	Geraniol	Cinnamal-dehyde	Anise Alcohol	Cinnamyl Alcohol	Eugenol
**G1**	0.022 ± 0.001			0.18 ± 0.06	0.14 ± 0.05	2.2 ± 0.6	0.4 ± 0.1	7 ± 1	59 ± 14		71 ± 21	
**G2**		1.2 ± 0.1	2.3 ± 0.2	143 ± 5	0.27 ± 0.04	0.179 ± 0.001	2.7 ± 0.4	3.2 ± 0.3			1.0 ± 0.3	
**G3**		0.11 ± 0.01	0.171 ± 0.002	1.66 ± 0.01	0.41 ± 0.01	0.12 ± 0.02	0.21 ± 0.01					
**G4**		3.0 ± 0.7			2.1 ± 0.5							
**G5**	0.89 ± 0.01	6.8 ± 0.5	10.2 ± 0.87	15 ± 1		53 ± 3	98 ± 2	172 ± 12	0.227 ± 0.001			26 ± 3
**G6**		0.66 ± 0.03	0.67 ± 0.01	1.4 ± 0.016	0.65 ± 0.01					11 ± 1	159 ± 14	
**G7**	0.19 ± 0.01	12 ± 1	11.7 ± 0.8	1.75 ± 0.08	2.58 ± 0.02	7.6 ± 0.9	36 ± 4	42 ± 5	0.17 ± 0.02			0.19 ± 0.02
**G8**	0.08 ± 0.01	4 ± 1	4.5 ± 1.6		1.37 ± 0.01	6.8 ± 0.1	3.8 ± 0.4	3.6 ± 0.2	0.14 ± 0.01		2.5 ± 0.1	
**G9**	1.0 ± 0.2	29 ± 9	28 ± 7	15 ± 4	4 ± 1		25 ± 7	217 ± 44	5.6 ± 0.5			6 ± 2
**G10**		0.7 ± 0.1	0.9 ± 0.1	36.2 ± 0.4	2.96 ± 0.04	29 ± 0.5	2.9 ± 0.1	26 ± 1				0.63 ± 0.08
**G11**		0.25 ± 0.05	0.22 ± 0.02	16 ± 2					0.18 ± 0.01		0.46 ± 0.08	
**G12**												
**G13**	0.83 ± 0.05	112 ± 4	121 ± 3	75 ± 2	40 ± 1	15 ± 3	41 ± 1	41 ± 1	0.21 ± 0.01			
**G14**		0.54 ± 0.01	0.47 ± 0.05	0.18 ± 0.04								
**G15**	0.27 ± 0.06	36 ± 11	37 ± 7	1.61 ± 0.09	0.19 ± 0.01		0.27 ± 0.02	2.1 ± 0.5				
**G16**						0.72 ± 0.02						
**G17**	0.3 ± 0.1	29 ± 8	26 ± 5	50 ± 10	3.2 ± 0.4	1.4 ± 0.2	5.6 ± 0.6	50 ± 7				
**G18**	1.94 ± 0.01	9.3 ± 1.2	9 ± 1	178 ± 2	12.2 ± 0.1			603 ± 15	7.20 ± 0.04	489 ± 14		
**G19**		0.61 ± 0.04	0.59 ± 0.08	0.27 ± 0.04						3.7 ± 0.2		
**G20**	0.97 ± 0.03	160 ± 2	181 ± 3	4.9 ± 0.2	0.82 ± 0.08			12.3 ± 0.3				
**G21**		0.14 ± 0.04	0.17 ± 0.02		1.6 ± 0.2							
**G22**		0.60 ± 0.06	0.300 ± 0.008			5.0 ± 0.3						
**G23**	4.3 ± 0.9	44 ± 7	57 ± 7		72 ± 11	0.12 ± 0.02		6 ± 1			13 ± 2	
**G24**		38 ± 3	40 ± 3	42 ± 5	45 ± 4	13 ± 1	102 ± 5	27 ± 5	1.6 ± 0.1			
**G25**						0.008 ± 0.001			0.30 ± 0.03			
**G26**												
**G27**												
**G28**	2.7 ± 0.4	9 ± 1	10 ± 1		1.1 ± 0.1	0.09 ± 0.01	1.1 ± 0.1	10 ± 2			5.5 ± 0.1	
**G29**		25 ± 2	23 ± 1	209 ± 17	3.31 ± 0.02	1.04 ± 0.02	7.27 ± 0.07				0.6 ± 0.1	
**G30**	3.6 ± 0.7	14 ± 1	13 ± 1	185 ± 25	13 ± 2		17 ± 1	1747 ± 145		17,036 ± 1039	327 ± 39	
**G31**						1.19 ± 0.06						
**G32**		65 ± 5	58 ± 3	115 ± 17								
**G33**	0.438 ± 0.063	23.3 ± 3.3	25 ± 3	52 ± 3	0.791 ± 0.007	38 ± 3	12 ± 0.4	18 ± 3				7.1 ± 0.2
**G34**				0.0363 ± 0.0025			0.29 ± 0.04	0.069 ± 0.002	0.90 ± 0.01	0.076 ± 0.002		
**G35**		0.228 ± 0.072	0.179 ± 0.016									
**G36**	2.7 ± 0.19	176 ± 7	212 ± 20	86 ± 5	1.768 ± 0.005	0.92 ± 0.06	37.8 ± 0.5	20.8 ± 0.79	2.5 ± 0.1	20.00 ± 0.02		20.0 ± 0.6
**G37**		0.07 ± 0.01	0.057 ± 0.006									
**G38**	0.28 ± 0.04	7 ± 2	8 ± 2	2.98 ± 0.01	0.135 ± 0.001	0.593 ± 0.008	5.0 ± 0.3	7.0 ± 0.1				
**G39**		1.0 ± 0.1	0.54 ± 0.04								3.7 ± 0.1	0.030 ± 0.007
**G40**		0.18 ± 0.024	0.10 ± 0.016									
**G41**	2.3 ± 0.3	144 ± 21	167 ± 24	3.6 ± 0.3	0.51 ± 0.04	10.6 ± 0.6	7.7 ± 0.4	7.7 ± 0.53	0.17 ± 0.02			0.26 ± 0.013
**G42**	0.7 ± 0.23	4.2 ± 0.3	3.8 ± 0.3	0.15 ± 0.01		2.73 ± 0.10	0.37 ± 0.02					
**G43**	0.032 ± 0.004	0.4 ± 0.1	0.42 ± 0.08	1.12 ± 0.03		0.51 ± 0.02	0.54 ± 0.01	1.02 ± 0.01		0.7 ± 0.2	1.51 ± 0.08	
**G44**		0.7 ± 0.23	0.7 ± 0.2									
**G45**		0.28 ± 0.01	0.22 ± 0.02				0.20 ± 0.03					
**G46**		0.07 ± 0.02	0.05 ± 0.02									
**G47**												
**G48**		0.32 ± 0.10	0.31 ± 0.02	0.04 ± 0.01		0.01 ± 0.01	0.22 ± 0.02					
**G49**							0.20 ± 0.01					
**G50**		0.44 ± 0.02	1.5 ± 0.19	11 ± 1	0.07 ± 0.01	42 ± 2	16.4 ± 0.7	15.9 ± 0.74		25.1 ± 1.5	2.0 ± 0.17	21 ± 1.
**G51**		0.48 ± 0.08	5.0 ± 0.7	9.5 ± 1.8		42 ± 7	26.2 ± 3.7	25 ± 3		259 ± 32	16 ± 1	82 ± 6
**G52**	0.14 ± 0.01	0.239± 0.06	0.26 ± 0.08			4.3 ± 1.1		0.325 ± 0.051				
**G53**		0.42 ± 0.09	0.48 ± 0.04	14.4 ± 1.8	0.74 ± 0.03	16.5 ± 2.1	8.725 ± 0.041	37.6 ± 4.1			156 ± 20	110 ± 28
**G54**										3.7 ± 1.1		
**G55**		0.44 ± 0.02	0.24 ± 0.02			3.06 ± 0.17						
**G56**		0.056 ± 0.008	0.057 ± 0.003			0.87 ± 0.13			0.658 ± 0.045			
**G57**	0.130 ± 0.023	0.218 ± 0.0010	0.12 ± 0.02							0.82 ± 0.12	0.28 ± 0.04	0.02 ± 0.01
**G58**		0.067 ± 0.0053	0.093 ± 0.0072	0.177 ± 0.001								
**G59**					0.31 ± 0.07			1.71 ± 0.50				
**G60**	0.117± 0.03	19 ± 6	27 ± 8	0.16 ± 0.04	0.22 ± 0.08			0.13 ± 0.02			0.3 ± 0.1	
**G61**												
**G62**	0.85 ± 0.07	74.7 ± 0.8	73.78 ± 0.29	17.9 ± 1.6		4.3 ± 0.4	12.2 ± 0.3	87 ± 11	0.23 ± 0.03		200 ± 5	
**G63**		0.12 ± 0.01	0.08748 ± 0.00040	0.096 ± 0.008		1.94 ± 0.017						
**G64**				0.19 ± 0.03	2.423 ± 0.001	0.029 ± 0.007						
**G65**		0.24 ± 0.02	2.9 ± 0.4		12.6 ± 0.7	0.97 ± 0.01	0.9 ± 0.12	0.75 ± 0.08		22 ± 8	18 ± 3	
**G66**		0.069 ± 0.003	0.04 ± 0.004		0.188 ± 0.001	1.72 ± 0.04	0.20 ± 0.02	0.15 ± 0.04		12 ± 2	0.17 ± 0.05	
**G67**	0.095 ± 0.0098	1.2 ± 0.1	1.0 ± 0.07	0.10 ± 0.01	0.08 ± 0.01							
	**Methyl Eugenol**	**Isoeugenol**	**α-Isomethyl ionone**	**Lilial**	**Amyl Cinnamal**	**Amylcinna-myl Alcohol**	**Farnesol**	**Hexyl Cinnamal**	**Benzyl Benzoate**	**Benzyl Salicylate**	**Benzyl Cinnamate**	**Total Content**
**G1**		2.2 ± 0.5	2.5 ± 0.6	0.17 ± 0.045		0.89 ± 0.27	9 ± 2		18 ± 56	0.9 ± 0.2	0.3 ± 0.10	173
**G2**			0.03 ± 0.01	0.0119 ± 0.001					0.5 ± 0.1	0.04 ± 0.01	0.020 ± 0.003	156
**G3**									0.30 ± 0.01	0.034 ± 0.002	0.019 ± 0.001	3.06
**G4**		2.37 ± 0.05	0.44 ± 0.07			11 ± 1			0.7 ± 0.2			20.2
**G5**	4.7 ± 0.5	1.17 ± 0.04	0.9 ± 0.1						0.19 ± 0.01			391
**G6**			0.045 ± 0.004	0.45 ± 0.02			0.061 ± 0.008	0.12 ± 0.01	0.23 ± 0.03	0.077 ± 0.002		174
**G7**			2.2 ± 0.3	4.91 ± 0.02			2.78 ± 0.02	1.1 ± 0.1	0.65 ± 0.05			126
**G8**								1.03 ± 0.09				28.4
**G9**		3.6 ± 0.8	1.7 ± 0.3	6.0 ± 0.4					22 ± 5	4 ± 1		369
**G10**		1.43 ± 0.09	9.95 ± 0.05	7.12 ± 0.02		0.53 ± 0.09	10.8 ± 0.3		9.9 ± 0.5	5.0 ± 0.8		146
**G11**												17.1
**G12**												0
**G13**									0.09 ± 0.03			448
**G14**												1.17
**G15**								1.4 ± 0.2	0.74 ± 0.02			80.2
**G16**												0.725
**G17**			7.8 ± 0.3		4180 ± 438		32 ± 4	149 ± 26	31 ± 4	0.13 ± 0.01		4567
**G18**	0.05 ± 0.01		0.06 ± 0.01					0.6 ± 0.3				1311
**G19**		4.0 ± 0.1	0.135 ± 0.004	0.0066 ± 0.0004	0.74 ± 0.11			0.4 ± 0.2	64 ± 1			74.7
**G20**		0.208 ± 0.010		517 ± 53		0.290 ± 0.009			0.28 ± 0.02	5.5 ± 0.9	0.21 ± 0.01	884
**G21**	0.06 ± 0.02		2.3 ± 0.6		0.33 ± 0.05	77.92 ± 0.07	4990 ± 5	0.14 ± 0.04		0.11 ± 0.06	0.10 ± 0.03	5073
**G22**												5.90
**G23**	0.08 ± 0.01				0.0341 ± 0.0082		5.25 ± 0.85	0.01 ± 0.03		0.28 ± 0.09		202
**G24**	0.092 ± 0.002		0.06 ± 0.01	15 ± 1		127 ± 1	28,737 ± 1		0.27 ± 0.05	0.06 ± 0.02	0.06 ± 0.015	29,190
**G25**				0.08 ± 0.01				0.18 ± 0.02	0.115 ± 0.008			0.640
**G26**								0.076 ± 0.005	0.052 ± 0.007	0.134 ± 0.005		0.263
**G27**										0.07 ± 0.01	0.11 ± 0.01	0.187
**G28**	0.09 ± 0.014	108 ± 1						0.292 ± 0.006	0.29 ± 0.01			149
**G29**	0.115 ± 0.005	1.004 ± 0.001	1.00 ± 0.03	10.12 ± 0.05				0.053 ± 0.009	9.912 ± 0.008	0.064 ± 0.004	0.050 ± 0.001	292
**G30**	0.047 ± 0.005	0.110 ± 0.005	0.11 ± 0.01									19,358
**G31**		0.65 ± 0.04										1.83
**G32**												238
**G33**			0.45 ± 0.03		0.05 ± 0.00	0.6 ± 0.1		46 ± 6	29 ± 3	0.345 ± 0.002		256
**G34**		1.3 ± 0.19	0.005 ± 0.001				1.8 ± 0.3	0.019 ± 0.001	0.055 ± 0.001			4.06
**G35**		8.01 ± 0.013						0.12 ± 0.01	0.06 ± 0.019			8.60
**G36**	0.20 ± 0.02	739 ± 7	0.018 ± 0.003		0.115 ± 0.002	0.036 ± 0.001	1427 ± 21	0.52 ± 0.03	0.22 ± 0.01			2748
**G37**												0.135
**G38**		0.55 ± 0.01	0.102 ± 0.001	9.03 ± 0.03	0.106 ± 0.003							40.8
**G39**		0.80 ± 0.02					0.71 ± 0.08	0.016 ± 0.003	0.053 ± 0.008			6.95
**G40**								0.016 ± 0.01				0.306
**G41**	0.011 ± 0.003					0.087 ± 0.001		6.3 ± 0.2	1.6 ± 0.29			353
**G42**			0.008 ± 0.001	0.0252 ± 0.00067	0.059 ± 0.002			0.382 ± 0.005	0.49 ± 0.03	0.142 ± 0.005		13.2
**G43**			0.07 ± 0.01	0.018 ± 0.005	0.67 ± 0.05	0.193 ± 0.003	1.40 ± 0.03	0.70 ± 0.05	0.55 ± 0.04		0.0268 ± 0.0076	10.0
**G44**					0.01 ± 0.00			0.014 ± 0.001	0.132 ± 0.001			1.63
**G45**				0.0068 ± 0.00042	0.027 ± 0.005			0.24 ± 0.01	0.40 ± 0.01			1.39
**G46**					0.011 ± 0.001			0.027 ± 0.004	0.13 ± 0.064			0.296
**G47**												0
**G48**			0.009 ± 0.002	0.05 ± 0.010	0.009 ± 0.002			0.02 ± 0.01	0.23 ± 0.04			1.32
**G49**									0.17 ± 0.03		0.13 ± 0.022	0.580
**G50**		2.0 ± 1.1	3.45 ± 0.44	0.0212 ± 0.0072	0.740 ± 0.065	0.082± 0.044		2.743 ± 0.003	1.905 ± 0.051	0.62 ± 0.07	0.05 ± 0.01	147
**G51**	0.0227 ± 0.0015	23.5 ± 1.9	65.4 ± 7.2	0.082 ± 0.011	32,458 ± 41	2.71 ± 0.87		755 ± 75		154 ± 19	0.836 ± 0.004	33,925
**G52**												5.23
**G53**	0.123 ± 0.011		130 ± 1	0.008 ± 0.001	1.1 ± 0.1	85 ± 16	6010 ± 163	0.23 ± 0.06	0.32 ± 0.02	0.38 ± 0.12		6573
**G54**							0.47 ± 0.02		1.2 ± 0.37	3.3 ± 1.9		8.63
**G55**								0.15 ± 0.02	0.08 ± 0.01	0.070 ± 0.006		4.05
**G56**	0.014 ± 0.005	0.031 ± 0.001		0.02 ± 0.01	0.10 ± 0.02			0.10 ± 0.02	0.16 ± 0.01	0.139 ± 0.01		2.21
**G57**			0.035 ± 0.001									1.63
**G58**				0.019 ± 0.008								0.358
**G59**							34 ± 13					36.1
**G60**												48.0
**G61**			0.012 ± 0.002							0.040 ± 0.004		0.0523
**G62**		135 ± 3		0.03 ± 0.001	0.039 ± 0.003	427 ± 4			259 ± 1	0.08 ± 0.01		1293
**G63**				0.08 ± 0.01				0.175 ± 0.006	0.11 ± 0.02	0.11 ± 0.01		2.75
**G64**				0.032 ± 0.001								2.68
**G65**		141 ± 11	0.029 ± 0.001		0.18 ± 0.04	0.75 ± 0.06	1274 ± 85	0.116 ± 0.009	0.10 ± 0.01	27.0 ± 0.01	0.028 ± 0.007	1502
**G66**		80 ± 9	0.014 ± 0.002		0.15 ± 0.07	0.007 ± 0.003	3.21 ± 0.01	0.19 ± 0.094	0.19 ± 0.056	93.44 ± 0.03	0.04 ± 0.02	191
**G67**				0.036 ± 0.005			0.9 ± 0.3					3.39

**Table 3:** Individual concentration (μg g^−1^) of the synthetic musks detected in 67 hydroalcoholic gel samples and total content.

	Musk Xylene	Galaxolide	Phantolide	Cashmeran	Traseolide	Tonalide	Ambrettolide	Total Content
**G1**		16 ± 4					4 ± 1	20
**G2**				0.16 ± 0.010				0.16
**G3**				0.25 ± 0.055				0.25
**G4**		0.38 ± 0.060		19 ± 3				19
**G5**		0.12 ± 0.01		0.56 ± 0.07				0.69
**G6**		0.17 ± 0.011		0.17 ± 0.02				0.31
**G7**		32 ± 4		0.13 ± 0.02				33
**G8**				0.23 ± 0.028				0.23
**G9**		32 ± 5			1.8 ± 0.3			34
**G10**								0
**G11**				0.10 ± 0.02				0.10
**G12**		0.10 ± 0.01						0.10
**G13**		0.08 ± 0.01					0.005 ± 0.001	0.087
**G14**		0.063 ± 0.001						0.063
**G15**								0
**G16**								0
**G17**		0.077 ± 0.004						0.077
**G18**		0.14 ± 0.01						0.14
**G19**		1.26 ± 0.07				0.036 ± 0.002		1.30
**G20**		2.5 ± 0.2					0.33 ± 0.011	2.9
**G21**		0.035 ± 0.002		0.128 ± 0.001				0.16
**G22**								0
**G23**		0.048 ± 0.003						0.048
**G24**		0.9 ± 0.1		0.38 ± 0.03				1.36
**G25**				0.053 ± 0.007				0.0053
**G26**								0
**G27**								0
**G28**		0.37 ± 0.04				0.08 ± 0.004		0.40
**G29**		39 ± 1		0.004 ± 0.001		0.38 ± 0.01		40
**G30**				0.011 ± 0.001				0.0111
**G31**								0
**G32**								0
**G33**		56 ± 6	0.14 ± 0.01	0.31 ± 0.04		0.15 ± 0.03		57
**G34**		0.043 ± 0.005		0.45 ± 0.05				0.49
**G35**								0
**G36**		0.44 ± 0.02		0.091 ± 0.006				0.54
**G37**								0
**G38**		35.12 ± 0.02	0.010 ± 0.001			0.094 ± 0.001		35.2
**G39**		0.032 ± 0.0039						0.0332
**G40**				0.0370 ± 0.0005				0.0370
**G41**			0.017 ± 0.001					0.0172
**G42**		1.69 ± 0.018						1.692
**G43**		0.09 ± 0.014						0.0910
**G44**		0.10 ± 0.013						0.102
**G45**								0
**G46**								0
**G47**								0
**G48**		0.14 ± 0.022						0.140
**G49**								0
**G50**		0.241 ± 0.001		0.17 ± 0.02				0.415
**G51**		8.7 ± 0.8		2.8 ± 0.2				11.6
**G52**				0.013 ± 0.001				0.0133
**G53**		0.017 ± 0.007	28.9 ± 0.6	0.8 ± 0.1				29.8
**G54**		0.056 ± 0.001						0.0567
**G55**		0.26 ± 0.04						0.266
**G56**		0.18 ± 0.02						0.188
**G57**		0.19 ± 0.098		0.008 ± 0.001				0.0202
**G58**		0.033 ± 0.001		0.014 ± 0.005				0.0471
**G59**		10 ± 3						10.4
**G60**								0
**G61**		0.613 ± 0.006						0.6133
**G62**								0
**G63**		0.31 ± 0.06				0.0220 ± 0.0003		0.332
**G64**		0.062 ± 0.003						0.0621
**G65**	2356 ± 31	0.05 ± 0.01						2355
**G66**	1840 ± 102	0.033 ± 0.001						1840
**G67**		0.077 ± 0.001		74.3 ± 0.2				74.4

**Table 4:** Individual concentration (μg g^−1^) of the preservatives detected in 67 hydroalcoholic gel samples and total content.

	PhEtOH	BHA	BHT	TCS	MeP	EtP	iPrP	PrP	iBuP	BzP	Total Content
**G1**			0.29 ± 0.01	0.3 ± 0.1		50 ± 11			1.2 ± 0.34		51.8
**G2**			0.012 ± 0.001								0.0121
**G3**			0.009 ± 0.001								0.0099
**G4**			0.05 ± 0.01		23 ± 4	29 ± 3		3.92 ± 0.02	61 ± 1		118
**G5**					2.5 ± 0.27	7.2 ± 0.9					9.84
**G6**						1.0 ± 0.1					1.06
**G7**						1.8 ± 0.1					1.81
**G8**	72.5 ± 0.5					2.3 ± 0.45					74.8
**G9**					2.5 ± 0.8						2.54
**G10**					1.19 ± 0.01			150 ± 13			151
**G11**						1.0 ± 0.1					1.0
**G12**						0.6 ± 0.1					0.654
**G13**	245 ± 2		1.15 ± 0.01	0.04 ± 0.01		2.6 ± 0.24					248
**G14**											0
**G15**	166 ± 15					0.58 ± 0.048					167
**G16**						0.64 ± 0.05			4.5 ± 0.2		5.16
**G17**		0.105 ± 0.002				62 ± 5			28 ± 3		90.8
**G18**									12.4 ± 0.8		12.4
**G19**				0.10 ± 0.0010	2.57 ± 0.13	0.76 ± 0.03			23 ± 2		26.4
**G20**									3.6 ± 0.1		3.62
**G21**				0.169 ± 0.003		40 ± 11			1.7 ± 0.2		42.3
**G22**	10,477 ± 797										10,477
**G23**							0.6 ± 0.2				0.644
**G24**			170 ± 1	0.03 ± 0.01	1.05 ± 0.07	10 ± 1			48.81 ± 0.04	2.9 ± 0.30	233
**G25**	0.5 ± 0.10										0.551
**G26**											0
**G27**				0.097 ± 0.005		3.4 ± 0.7					3.53
**G28**			0.034 ± 0.006								0.0348
**G29**		0.12 ± 0.01	6.19 ± 0.04	0.088 ± 0.007	0.49 ± 0.015	4.24 ± 0.01			0.45 ± 0.01		11.6
**G30**					0.18 ± 0.01	1.5 ± 0.25					1.73
**G31**	9352 ± 19										9352
**G32**				0.136 ± 0.001							0.0136
**G33**	1.217 ± 0.098				5.4 ± 0.4		1.0 ± 0.19				7.71
**G34**	65 ± 36					1.3 ± 0.2	0.3 ± 0.1				66.6
**G35**	7908 ± 815										7908
**G36**	16.3 ± 4.4		2.902 ± 0.076		1.142 ± 0.097	3.6 ± 0.2					24.0
**G37**						4 ± 1					3.8
**G38**		0.260 ± 0.002	0.0125 ± 0.0010			6.3 ± 0.4					6.57
**G39**	3196 ± 871					0.8 ± 0.1					3197
**G40**											0
**G41**	0.55 ± 0.12										0.551
**G42**	7.0 ± 2.0					5.94 ± 0.04			0.243 ± 0.008		13.2
**G43**	0.212 ± 0.065					1.6 ± 0.3					1.85
**G44**			0.056 ± 0.007			4.4 ± 0.5	0.13 ± 0.013				4.60
**G45**						3.1 ± 0.4					3.18
**G46**	0.6 ± 0.11										0.61
**G47**											0
**G48**	1.6 ± 0.3					6.3 ± 0.87					7.96
**G49**	13,735 ± 373			0.454 ± 0.001				0.151 ± 0.006			13,735
**G50**	19 ± 1	0.12 ± 0.02	0.003 ± 0.001		1.9 ± 0.7	15 ± 2					36.4
**G51**	21 ± 3	0.57 ± 0.04	0.020 ± 0.002	0.32 ± 0.09	22 ± 2			0.3 ± 0.11			44.5
**G52**	28 ± 7										28.8
**G53**	8 ± 1	0.35 ± 0.07	0.8 ± 0.1	0.05 ± 0.01	7.0 ± 0.92				13.1 ± 0.6		29.7
**G54**						9 ± 4				17.3 ± 0.2	26.0
**G55**	20.0 ± 6.5				0.61 ± 0.07	5.4 ± 0.2					26.1
**G56**	5428 ± 104				1.21 ± 0.05	8 ±1				59 ± 15	5495
**G57**						3.4 ± 0.1					3.46
**G58**			0.008 ± 0.002								0.0086
**G59**						1.7 ± 0.5					1.76
**G60**											0
**G61**			0.014 ± 0.001								0.0145
**G62**					1.3 ± 0.10					4.68 ± 0.14	6.02
**G63**	1.00 ± 0.069	0.0124 ± 0.0003	0.008 ± 0.001		0.50 ± 0.08						1.54
**G64**											0
**G65**				0.05 ± 0.017			0.13 ± 0.04		0.7 ± 0.3		0.942
**G66**				0.05 ± 0.02			0.070 ± 0.003		0.14 ± 0.05		0.302
**G67**			4.3 ± 0.3			15 ± 3					19.3

**Table 5:** Individual concentration (μg g^−1^) of plasticisers detected in 67 hydroalcoholic gel samples and total content.

	DMP	DEP	DPP	DEHP	DMA	DEA	DEHA	Total Content
**G1**				0.4 ± 0.13	0.22 ± 0.01	1.0 ± 0.3		1.73
**G2**		1.70 ± 0.07			0.10 ± 0.01			1.82
**G3**		0.230 ± 0.0034		0.12 ± 0.03	0.22 ± 0.04			0.586
**G4**				0.15 ± 0.02				0.155
**G5**	0.42 ± 0.05			0.114 ± 0.004	4.0 ± 0.1			4.61
**G6**	0.59 ± 0.010	25.5 ± 0.92						26.1
**G7**		3.2 ± 0.2						3.25
**G8**		0.19 ± 0.02						0.191
**G9**	10 ± 2	104 ± 7						113.8
**G10**		1.8 ± 0.2						1.87
**G11**								0
**G12**								0
**G13**	0.45 ± 0.010	1804 ± 123				0.15 ± 0.013		1804
**G14**								0
**G15**								0
**G16**								0
**G17**		8.7 ± 1.2						8.7
**G18**								0
**G19**	0.53 ± 0.07			1.1 ± 0.1		1.37 ± 0.02		3.09
**G20**		8238 ±783					0.399 ± 0.007	8238
**G21**								0
**G22**								0
**G23**		0.68 ± 0.30						0.68
**G24**								0
**G25**								0
**G26**								0
**G27**								0
**G28**	108 ± 4					0.098 ± 0.01		108
**G29**		6.3 ± 0.3				0.11 ± 0.01		6.51
**G30**						0.047 ± 0.0012		0.047
**G31**	0.63 ± 0.09	4888 ± 13						4888
**G32**		6.5 ± 0.7						6.5
**G33**								0
**G34**		0.19 ± 0.05			0.7 ± 0.1			0.85
**G35**								0
**G36**		0.24 ± 0.03		0.14 ± 0.036	0.56 ± 0.027			0.96
**G37**								0
**G38**		8 ± 1		0.25 ± 0.045				8.58
**G39**		0.2 ± 0.1		0.14 ± 0.04	0.212 ± 0.005	0.010 ± 0.007		0.569
**G40**		0.231 ± 0.007						0.231
**G41**								0
**G42**								0
**G43**								0
**G44**				0.9 ± 0.2		0.053 ± 0.001		0.957
**G45**				2.8 ± 0.2				2.8
**G46**								0
**G47**								0
**G48**								0
**G49**								0
**G50**		0.79 ± 0.05						0.79
**G51**	0.16 ± 0.01	4 ± 1		2.1 ± 0.3		0.080 ± 0.001	0.9 ± 0.2	7.79
**G52**								0
**G53**		1910 ± 106		12 ± 3				1922
**G54**								0
**G55**								0
**G56**		0.6 ± 0.1	0.11 ± 0.02	0.19 ± 0.05				0.952
**G57**								0
**G58**								0
**G59**								0
**G60**								0
**G61**				0.24 ± 0.02				0.24
**G62**		538 ± 4						538
**G63**				0.333 ± 0.094				0.333
**G64**								0
**G65**			0.05 ± 0.01					0.050
**G66**			0.14 ± 0.06			1.77 ± 0.08		1.92
**G67**								0

## Data Availability

All data are available in the manuscript and Supplementary Material.

## Supplementary Materials

The following supporting information can be downloaded at: https://www.mdpi.com/article/10.3390/mps6050095/s1, Table S1: Target compounds. CAS number, molecular mass, retention time, MS/MS transitions and restrictions for leave-on cosmetics (EC 1223/2009 regulation) [6]; Table S2: Information for the analysed samples including the composition indicated on the label; Figure S1: Comparison of the recoveries obtained for fortified hydroalcoholic gel samples free of target compounds (except DEP) at two levels: 0.2 µg g^−1^ and 2 µg g^−1^. Table S3: SPME-GC-MS/MS performance. Linearity, precision, recoveries, instrumental detection limits (IDLs) and limits of detection (LODs).
