# Supplementary material for: Allergens and Other Harmful Substances in Hydroalcoholic Gels: Compliance with Current Regulation
Source: Methods Protoc. 2023 Oct 7;6(5):95. doi: 10.3390/mps6050095 (PMC10609030; doi:10.3390/mps6050095)

Table S1. Target compounds. CAS number, molecular mass, retention time, MS/MS transitions and restrictions for leave-on cosmetics (EC 1223/2009 regulation) [6].

Compound	CAS number	Molecular mass	Retention time	MS/MS transition (collision energy, eV)[a]	Restrictions[b]
Fragrance allergens					
Pinene	80-56-8	136	4.28	77.1 → 50.8 (15)<br>92.8 → 39.0 (35)<br>92.8 → 77.0 (10)	
Limonene	5989-27-5	136	6.03	67.9 → 53.0 (10)<br>92.8 → 77.0 (15)<br>92.8 → 91.0 (10)	0.001%[c]
Benzyl alcohol	100-51-6	108	6.10	79.0 → 50.9 (20)<br>107.1 → 79.1 (10)<br>108.2 → 79.1 (15)	0.001%[d]<br>1.0%[d]
Linalool	78-70-6	154	7.13	71.0 → 43.0 (10)<br>92.9 → 77.0 (10)<br>92.9 → 91.0 (10)	0.001%[c]
Methyl-2-octynoate	111-12-6	154	8.34	79.0 → 77.0 (10)<br>94.9 → 67.0 (10)<br>122.9 → 67.1 (10)	0.001%[c]<br>0.01%[e]
Citronellol	106-22-9	156	8.61	67.1 → 41.0 (15)<br>95.0 → 67.1 (10)<br>123.1 → 81.1 (10)	0.001%[c]
Citral	5392-40-5	152	8.84	68.9 → 41.0 (10)<br>94.0 → 79.0 (10)<br>108.9 → 81.0 (10)	0.001%[c]
Geraniol	106-24-1	154	8.84	92.9 → 51.0 (25)<br>92.9 → 77.1 (10)<br>110.9 → 93.1 (5)	0.001%[c]
Cinnamaldehyde	104-55-2	132	9.13	103.1 → 77.0 (10)<br>131.1 → 51.0 (40)<br>131.1 → 77.0 (25)	0.001%[c]
Anise alcohol	105-13-5	138	9.20	109.1 → 77.0 (15)<br>109.1 → 94.0 (10)<br>137.0 → 77.0 (20)	0.001%[c]
Cinnamyl alcohol	104-54-1	134	9.40	92.1 → 91.0 (10)<br>134.1 → 78.0 (15)<br>134.1 → 91.2 (20)	0.001%[c]
Eugenol	97-53-0	164	9.72	131.1 → 103.0 (10)<br>164.2 → 103.0 (20)	0.001%[c]
Methyleugenol	93-15-2	178	10.02	147.0 → 91.1 (10)<br>163.0 → 107.1 (10)<br>178.1 → 147.1 (10)	
Isoeugenol	97-54-1	164	10.03	77.0 → 50.9 (15)<br>103.0 → 77.0 (10)<br>164.1 → 149.1 (10)	0.001%[c]<br>0.02%[f]
α-isomethylionone	127-51-5	206	10.54	107.0 → 91.0 (10)<br>150.1 → 91.0 (20)<br>150.1 → 135.1 (10)	0.001%[c]
Lilial®	80-54-6	204	10.89	189.2 → 131.1 (10)<br>204.2 → 147.2 (10)<br>204.2 → 189.2 (10)	Prohibited[g]
Amylcinnamal	122-40-7	202	11.71	116.8 → 115.1 (10)<br>128.9 → 128.0 (20)<br>202.0 → 129.1 (10)	0.001%[c]
Amylcinnamyl alcohol	101-85-9	204	12.01	91.1 → 65.0 (15)<br>133.1 → 55.0 (10)<br>133.1 → 115.1 (10)	0.001%[c]
Farnesol	4602-84-0	222	12.03	69.0 → 39.0 (15)	0.001%[c]

				69.0 → 41.0	(10)	
Hexylcinnamal	101-86-0	216	12.56	116.8 → 91.0	(15)	0.001% [c]
				129.0 → 127.0	(20)	
				216.3 → 129.1	(10)	
Benzyl benzoate	120-51-4	212	12.87	90.9 → 65.0	(15)	0.01% [h]
				105.1 → 77.0	(10)	
				194.1 → 165.1	(20)	
Benzyl salicylate	118-58-1	228	14.14	91.0 → 39.0	(30)	0.001% [c]
				91.0 → 65.0	(15)	
				228.1 → 91.0	(10)	
Benzyl cinnamate	103-41-3	238	17.94	131.1 → 77.0	(20)	0.001% [c]
				131.1 → 103.0	(10)	
				192.0 → 191.2	(15)	
Synthetic musks						
Musk Xylene	81-15-2	297	13.80	282.0 → 265.1	(10)	0.03% [i]
				282.1 → 90.8	(35)	
Musk Ambrette	83-66-9	268	11.39	253.1 → 105.9	(10)	Prohibited [g]
				253.1 → 119.9	(10)	
				268.1 → 253.1	(5)	
Musk Moskene	116-66-5	278	14.08	263.1 → 127.8	(40)	Prohibited [g]
				263.1 → 200.9	(10)	
				263.1 → 221.1	(10)	
Musk Tibetene	145-39-1	266	14.84	251.1 → 159.9	(10)	Prohibited [g]
				266.3 → 251.2	(5)	
Galaxolide	1222-05-5	258	13.70	213.2 → 171.2	(10)	
				243.2 → 198.2	(20)	
				243.2 → 213.2	(10)	
Celestolide	13171-00-1	244	12.22	229.3 → 173.1	(10)	
				244.2 → 173.1	(15)	
				244.2 → 229.2	(10)	
Phantolide	15323-35-0	244	12.61	187.0 → 145.2	(10)	2% [j]
				229.2 → 187.0	(10)	
				244.2 → 229.2	(10)	
Cashmeran	33704-61-9	206	10.74	163.1 → 107.1	(10)	
				191.1 → 135.1	(10)	
				206.2 → 191.2	(10)	
Traseolide	68140-48-7	258	13.73	173.0 → 131.1	(10)	
				215.2 → 173.1	(10)	
				258.2 → 215.2	(10)	
Tonalide	1506-02-1	258	13.77	187.1 → 43.0	(15)	1% [k]
				243.2 → 159.2	(10)	
				258.2 → 187.2	(10)	
Ambrettolide	7779-50-2	252	15.02	95.9 → 81.1	(10)	
				108.9 → 67.1	(10)	
				108.9 → 81.0	(10)	
Preservatives						
Phenoxyethanol (PhEtOH)	122-99-6	138	8.59	94.0 → 65.1	(15)	1.0% [l]
				94.0 → 65.8	(10)	
				138.0 → 94.1	(10)	
Butylhydroxyanisole (BHA)	25013-16-5	180	10.60	121.0 → 93.1	(10)	
				152.0 → 121.1	(10)	
				137.1 → 77.1	(20)	
Butylhydroxytoluene (BHT)	128-37-0	220	10.70	165.2 → 137.1	(10)	0.8% [m]
				180.1 → 165.1	(10)	
				205.1 → 145.1	(15)	
Triclosan (TCS)	3380-34-5	290	18.00	138.0 → 121.1	(10)	
				218.0 → 126.9	(30)	
				288.0 → 218.0	(15)	
Methyl paraben (MeP)	99-76-3		10.44	152.0 → 121.1	(10)	
		152		121.0 → 93.1	(10)	0.4% [n]

				121.0 → 65.1 (15)	
Ethyl paraben (EtP)	120-47-8	166	10.86	121.0 → 93.1 (10)<br>138.0 → 121.0 (10)<br>121.0 → 65.1 (15)	0.4%[n]
Isopropyl paraben (iPrP)	4191-73-5	180	11.03	121.0 → 93.1 (10)<br>138.0 → 121.0 (10)<br>121.0 → 65.1 (15)	Prohibited[g]
Propyl paraben (PrP)	94-13-3	180	11.52	121.0 → 93.1 (10)<br>138.1 → 121.0 (10)<br>121.0 → 65.0 (15)	0.14%[o]
Isobutyl paraben (iBuP)	4247-02-3	194	11.97	138.0 → 121.0 (10)<br>121.1 → 65.1 (15)<br>121.1 → 93.1 (10)	Prohibited[g]
Benzyl paraben (BzP)	94-18-8	228	18.48	121.0 → 65.1 (20)<br>121.0 → 93.1 (10)<br>228.2 → 121.1 (10)	Prohibited[g]
Plasticizers					
Dimethyl phtalate (DMP)	131-11-3	194	10.37	163.0 → 77.1 (20)<br>163.0 → 133.1 (10)<br>163.0 → 135.1 (10)	
Diethyl phthalate (DEP)	84-66-2	222	11.26	149.0 → 121.1 (10)<br>176.7 → 149.1 (10)	
Diisobutyl phthalate (DIBP)	84-69-5	278	13.79	149.0 → 65.0 (25)<br>149.0 → 121.0 (15)<br>223.1 → 149.0 (10)	Prohibited[g]
Dibutyl phthalate (DBP)	84-74-2	278	15.27	149.0 → 65.1 (20)<br>149.0 → 93.0 (15)<br>149.0 → 121.0 (10)	Prohibited[g]
Dimethoxyethyl phthalate (DMEP)	117-82-8	282	15.94	104.2 → 76.0 (10)<br>148.9 → 65.0 (20)<br>148.9 → 121.0 (10)	Prohibited[g]
Diisopentyl phthalate (DIPP)	605-50-5	306	17.21	149.0 → 93.0 (15)<br>149.0 → 121.0 (15)<br>237.1 → 149.0 (10)	Prohibited[g]
Dipentyl phthalate (DPP)	131-18-0	306	18.46	149.0 → 65.0 (25)<br>149.0 → 93.0 (15)<br>149.0 → 121.0 (15)	Prohibited[g]
Benzylbutyl phthalate (BBP)	85-68-7	312	20.11	149.0 → 65.1 (20)<br>149.0 → 93.0 (15)<br>205.9 → 149.1 (10)	Prohibited[g]
Diisohetpty phthalate (DIHP)	41451-28-9	362	20.78	149.0 → 65.0 (25)<br>149.0 → 121.0 (15)<br>265.1 → 149.0 (10)	
Diethylhexyl phthalate (DEHP)	117-81-7	390	21.07	149.0 → 93.0 (15)<br>149.0 → 121.0 (15)<br>279.1 → 149.1 (10)	Prohibited[g]
Dicyclohexyl phthalate (DCHP)	84-61-7	330	21.03	149.0 → 65.0 (25)<br>149.0 → 93.0 (15)<br>249.1 → 149.1 (15)	Prohibited[g]
Diphenyl phthalate (DPhP)	84-62-8	318	21.18	225.0 → 77.0 (20)<br>225.0 → 115.0 (30)<br>225.0 → 152.8 (10)	
Di-n-octyl phthalate (DnOP)	117-84-0	390	22.20	149.0 → 93.0 (15)<br>149.0 → 121.0 (15)	
Dimethyl adipate (DMA)	627-93-0	174	8.74	111.0 → 83.0 (10)<br>114.0 → 71.0 (15)<br>114.0 → 113.0 (10)	
Diethyl adipate (DEA)	141-28-6	202	9.88	128.3 → 99.0 (10)<br>157.0 → 83.0 (15)<br>157.0 → 111.0 (5)	

Diethylhexyl adipate (DEHA)	103-23-1	370	20.31	129.0 → 83.0 (10)<br>129.0 → 101.0 (5)<br>146.9 → 101.0 (10)

[a] Underlined MS/MS transition is the selected for quantification.
[b] Cosmetic Regulation (EU) Nº 1223/2009. Current consolidated version: 17/12/2022.
[c] The presence of the substance must be indicated in the list of ingredients when its concentration exceeds: **0.001% in leave-on products** and 0.01% in rinse-off products.
[d] For purposes other than inhibiting the development of microorganisms in the fragrance/ aromatic compositions/ raw materials. the presence of the substance must be indicated in the list of ingredients when its concentration exceeds **0.001% in leave-on products** and 0.01% in rinse-off products. As a preservative. its maximum permitted concentration in final product is **1.0%.**
[e] Maximum permitted concentration in final product: **0.01%.**
[f] Maximum permitted concentration in final products except oral products: **0.02%.**
[g] Prohibited substances in cosmetic products are collected in the Annex II. The European Cosmetics legislation also prohibits the use of substances classified as carcinogenic. mutagenic and reprotoxic (CMR) hazards (Article 15).
[h] The presence of the substance must be indicated in the list of ingredients when its concentration exceeds: **0.01% in leave-on products** and in rinse-off products.
[i] All cosmetic products with the exception of oral products. Maximum permitted concentration in final products: 1.0% in fine fragrance. 0.4% in eau de toilette and **0.03% in other products**.
[j] Maximum concentration in **leave-on products: 2%** and no limit in rinse-off products.
[k] Maximum permitted concentration in final leave-on products: 0.1%. except **hydroalcoholic products 1%**. fine fragrance 2.5%. fragrance cream 0.5%. In rinse-off products: 0.2%.
[l] Maximum permitted concentration in final products: **1.0%.**
[m] Maximum permitted concentration in mouthwash is 0.001%. in toothpaste 0.1% and in other **leave-on** and rinse-off products **0.8%**.
[n] Maximum concentration is **0.4% (as acid) for single ester** and 0.8% (as acid) for mixtures of ester.
[o] Maximum concentration is **0.14% (as acid) for individual esters** and 0.8% (as acid) for mixtures of esters. where the sum of their individual concentrations does not exceed 0.14%. Not to be used in leave-on products designed for application on the nappy area of children under three years of age.

Table S2. Information of the analyzed samples including the composition indicated on the label.

Code	Sampling date and place	Name	Composition declared on the label
**G1**	9/11/2020 Personal use	Hands gel cleaner. Leave-on product.	Alcohol denat, water, polysorbate-20, propylene, glycol, **fragrance (parfum)**, aloe barbadensis leaf juice, glycerin, triethanolamine, carbomer,tocopheryl acetate (vitamin E acetate), hydroxypropylethyl cellulose, C.I. 14700.
**G2**	9/11/2020 Personal use	Sanitizer hydroalcoholic gel. Alcohol 70%. Hands cleaner without water.	Alcohol denat (70%), aqua (water), glycerin, acrylates / C 10-30 Alkyl Acrylate Crosspolymer, PEG-40 Hydrogenated Castor Oil, triethanolamine, CI 42090 (FD & Blue 1).

**G3**	9/11/2020 Supermarket	Sanitizer hydroalcoholic gel. 70% of alcohol.	Alcohol denat, aqua (water), isopropyl alcohol, glycerin, carbomer, triethanolamine.
**G4**	9/11/2020 Local store	Hydroalcoholic gel with aloe vera and algae.	Alcohol, aqua (water), isopropyl alcohol, glycerin, **parfum**, acrylates copolymer, aloe barbadensis leaf extract, triethanolamine, fucus vesiculosus extract, sodium benzoate, potassium sorbate, CI 42090, CI 19140, sodium chloride, citric acid.
**G5**	9/11/2020 Local store	Hands hydroalcoholic gel. 70% of alcohol.	Alcohol denat, aqua (water), glycerin, **phenoxyethanol**, aloe barbadensis leaf juice, didecyldimonium chloride, C.I. 42090.
**G6**	15/11/2020 Local store	Gel higienizante de manos sin aclarado. Alcohol 80%.	Alcohol denat, aqua (water), glycerin, ammonium polyacryloyldimethyltaurate, ethanolamine.
**G7**	15/11/2020 Bank	Hydroalcoholic gel. Antiseptic for healthy skin. Hands disinfectant.	Composition for 100 g:<br>Ethanol, 75 g.<br>Water and excipients, c.s.p. 100 g.
**G8**	17/11/2020 Personal use	Antiseptic hydroalcoholic gel for healthy skin.	75% (v/v) ethanol, 0.1% (w/w), C12-16-Benzyldimethyldecylammonium chloride, limonene, hexylcinnamal, hydroxycitronellal.
**G9**	17/11/2020 Personal use	Hydroalcoholic gel 75%. Hand sanitizer with aloe vera and without water	Alcohol, aqua (water), glycerin, carbopol, aloe, parfum, aminomethyl propanol.
**G10**	17/11/2020 Personal use	Hand cleaner. Hydroalcoholic gel.	Alcohol denat, aqua (water, eau), hydroxyethyl urea, parfum (fragrance), acrylates/C10-30 alkyl acrylate crosspolymer, ethanolamine, hexyl laurate, polysorbate 80, benzophenone-1, butylphenyl methylpropional (lilial), linalool, citronellol, hexyl cinnamal, benzyl salicylate, benzyl alcohol, ethyl benzoate, methyl benzoate.
**G11**	Restaurant (17/11/2020)	Hand sanitizer.	Alcohol, isopropyl alcohol, gliceryn, hydroxyethylcellulose.
**G12**	Personal use (04/10/2021)	Hydroalcoholic gel 70 %.	Alcohol, aqua, isopropyl alcohol, aloe barbadensis leaf juice, glycerin, carbomer, triethanolamine.
**G13**	Personal use (04/10/2021)	Hydroalcoholic gel for hands sanitising and disinfecting	Ethanol 54.3 g excipients and water c.s.p. 100 g. Limonene, linalool, citral, geraniol, hydroxycitronellal.

**G14**	Personal use (04/10/2021)	Hydroalcoholic product	Alcohol denat (70 %), aqua (water), glycerin, acrylates C10-30 Alkyl Acrylate C osspolymer, PEG-40 Hydrogenated Castor Oil, Triethanolamine, CI 42090 (FD & Blue 1).
**G15**	04/10/2021 Personal use	Antiseptic for healthy skin	Ethanol 75 % (v/v), 0.1 % (w/w) Benzyldimethyldecylammonium chloride, limonene, hexylcinnamal.
**G16**	Personal use (05/10/2021)	Hydroalcoholic solution	Alcohol denat 80 % (v/v), aqua, glycerin, hydrogen peroxide.
**G17**	Personal use (03/10/2021)	Hands gel. Antiseptic for healthy skin. Desinfectant.	Ethanol 78 % (w/w), hexylcinnamal, amylcinnamal, limonene, linalool, geraniol, citronellol.
**G18**	Personal use (03/10/2021)	Hydroalcoholic gel	Alcohol denat 72.5 % (v/v), aqua (water), glycerin, propylene glicol, ammonium polyacryloyldimethyl, taurate, parfum, limonene, linalool.
**G19**	Personal use (03/10/2021)	Hydroalcoholic solution	Alcohol denat, aqua (water), glycerin, benzalkonium chloride, parfum.
**G20**	Personal use (04/10/2021)	Hands gel cleaner	Alcohol denat, water, polysorbate-20, propyleneglycol, fragrance (parfum), aloe barbadensis leaf juice, glycerin, triethanolamine, carbomer, tocopheryl acetate (vitamin E acetate), hydroxypropylethyl cellulose.
**G21**	Hospital (07/12/2021)	Antiseptic for healthy skin. Hydroalcoholic gel	Ethanol (700 mg/g or 755 mL/L) in the presence of thickening agents, moisturisers, emollients and water.
**G22**	Shop (17/12/2021)	Antiseptic hydroalcoholic gel for healthy skin	Ethanol(70%), phenoxyethanol (2.1 %) ,didecyldimethylammonium chloride (0.35%) UNE-EN 1276, UNE-EN 1650, UNE-EN 14476, UNE-EN 12791, UNE-EN 13624, UNE-EN 14348, UNE-EN 13727
**G23**	Personal use (18/12/21)	Hygiene foam	Aqua (water), sodium laureth sulfate, sodium lauryl sulfate, citric acid, coco glucoside, glyceril oleate, citrus limon (lemon) peel oil, **limonene**, sodium benzoate, eucalyptus globulus leaf oil, copper sulfate, lactic acid, potassium sorbate, **citral**, tocopherol, hydrogenated palm glycerides citrate
**G24**	Car dealer (01/02/22)	Solution for skin cleaning	Alcohol, aqua, glycerin, propylene glycol, aloe barbadensis extract, parfum, carbomer, triethanolamine, tocopherol, citral, geraniol, benzyl benzoate, citronellol, limonene, linalool
**G25**	Optics (05/02/22)	Hand sanitiser	Alcohol denat, aqua, glycerin, carbomer, triethanolamine, aloe barbadensis leaf extract
**G26**	Shopping centre (05/02/22)	Hygienic hand rub	Ethanol 700 mL/L (556.5 g/L)
**G27**	Restaurant (03/02/22)	Hydroalcoholic solution	Ethanol (72% w/w)

**G28**	Optics (03/02/22)	Hydroalcoholic gel	Alcohol denat, water, aloe barbadensis leaf juice, glycerin, isopropyl alcohol, glyceryl cocoate, triethanolamine, fragrance (parfum), benzyl salicylate, hexyl cinnamal, limonene, linalool, potassium sorbate, sodium benzoate, sodium sulfite, acrylates/C10-30 alkyl acrylate crosspolymer
**G29**	Dentist (04/02/22)	Hand sanitiser	Alcohol denat, water, hydroxyethylcellulose, parfum, benzalkonium chloride, disodium phosphate, polysorbate 60, sodium phosphate, limonene, linalool
**G30**	Personal use (04/02/22)	Hydroalcoholic gel	Alcohol denat, water, glycerin, propylene glycol, ammonium polyacryloyldimethyl taurate, parfum, limonene, linalool
**G31**	Personal use (06/02/22)	Hydroalcoholic gel for hands sanitising.	Alcohol denat, water, aloe barbadensis, phenoxyethanol, acrylates/C10-30 alkyl acrylate crosspolymer, amonomehyl propanol. Benzalkonium chloride, chlorhexidine digluconate, glyceyl cocoate
**G32**	Post office (09/02/22)	Hygienic hands gel	Alcohol denat, aqua, acrylates/C10-30 alkyl acrylate crosspolymer, aloe barbadensis leaf juice, glycerin, isopropyl alcohol, PEG-7 glyceryl cocoate, triethanolamine, parfum, benzyl salicylate, hexyl cinnamal, limonene, linalool, potassium sorbate, sodium benzoate, sodum sulfite
**G33**	Shop (09/11/20)	Hand sanitizer	Alcohol denat, aqua, gliceryn, phenoxyethanol, aloe barbadensis leaf juice, didecyldimonium chloride, C.I. 42090. Absence of parfum
**G34**	Personal use (17/11/20)	Hydroalcoholig gel for hand antisepsis	Alcohol denat, aqua/water, glycerin, ammonium polyacryloyldimethyl taurate
**G35**	Restaurant (11/02/2022)	Hydroalcoholic lotion for healthy skin and pre-surgical hand disinfection	Ethanol 70% w/w, phenoxyethanol 2.1% w/w, didecyldimethylammonium chloride 0.35% w/w, UNE-EN 1276, UNE-EN 1650, UNE-EN 14476, UNE-EN 12791, UNE-EN 13624, UNE-EN 14348, UNE-EN 13727
**G36**	Personal use (15/02/2022)	No-rinse hand cleansing gel	Alcohol denat, aqua (water), glycerin, PEG-40 hydrogenated castor oil, acrylates/C10-30 alkyl acrylate crosspolymer, parfum (fragrance), tromethamine, limonene, PEG-30 dipolyhydroxustearate, trideceth-6, butyl methoxydibenzoylmethane, ethylhexyl methoxybezoylmethane, ethylhexyl methoxycinnamate, PPG-26-Buteth-26, citrus paradisi (grapefruit) juice, linalool, enchylhexyl salicylate, citral, potassium sorbate, Cl 14700 (Red 4), Cl 17200 (Red 33), Cl 19140 (Yellow 5), BHT.

**G37**	Restaurant (11/02/22)	Hydroalcoholic lotion for healthy skin and pre-surgical hand disinfection	Ethanol 70% w/w, phenoxyethanol 2.1% w/w, didecyldimethylammonium chloride 0.35% w/w,<br>UNE-EN 1500, UNE-EN 1276, UNE-EN 1650, UNE-EN 14476, UNE-EN 12791, UNE-EN 13624, UNE-EN 14348, UNE-EN 13727
**G38**	Shop (11/02/22)	Hydroalcoholic gel	Isopropyl alcohol (70% w/w), excipient, aqua (csp 100%).
**G39**	School (09/02/2022)	Hands hydroalcoholic gel	Aqua, alcohol, isopropyl alcohol, gliceryn, hydroxyethylcellulose
**G40**	Book store (16/02/2022)	Hands hydroalcoholic gel	Alcohol denat, aqua, glycerin, carbomer, propylene glycol, aloe barbadensis leaf extract, aminomethyl propanol, potassium sorbate, parfum
**G41**	Restaurant/dentist (17/02/2022)	Hand cleaner gel	Alcohol denat, aqua, glycerin, aloe barbadensis leaf juice, acrylates/C10-30 alkyl acrylate crosspolymer, parfum, citric acid, sodium benzoate, potassium sorbate, triethanolamine, limonene
**G42**	(05/03/2022)	Hydroalcoholic gel. Leave-on product.	Alcohol Denat, Aqua, Glycerin, Carbomer, Triethanolamine.
**G43**	Exhibition (06/03/2022) (25/03/2022)	Hydroalcoholic gel. Antiseptic. 80% Alcohol.	Ethanol 79,8% p/p
**G44**	Museum (06/03/2022)	Hydroalcoholic gel. Antiseptic for healthy skin. Sanitizer.	Ethanol 70% p/p, phenoxyethanol 2,1% w/w, didecyldimethylammonium chloride 0.35% w/w, excipients CSP 100%.<br>"Absence of parfum and dyes"<br>UNE-EN 1276, UNE-EN 1650, UNE-EN 14476, UNE-EN 12791, UNE-EN 13624, UNE-EN 14348, UNE-EN 13727
**G45**	Shopping centre (06/03/2022)	Hydroalcoholic gel. Antiseptic dermatological gel. Leave-on product.	"Equilibrada combinación de alcohols, potenciada con desinfectantes a base de biguanida y acondicionadores de la piel". Alcohol etílico, alcohol isopropílico, "exento de perfumes"
**G46**	Pharmacy (09/03/2022)	Hydroalcoholic gel. Antiseptic for healthy skin	Ethanol (62 %), isopropanol (10 %), Benzalkonium chloride (0.05 %), excipients, c.s.p. 100%.
**G47**	Personal use (19/03/2022)	Hydroalcoholic gel to prevent infections	70 % alcohol denat, water (aqua), glycerin, hydroxyethylcellulose
**G48**	Church/Shop (20/03/2022)	Sanitizing hydroalcoholic gel	Alcohol denat, aqua, isopropyl alcohol, neopentyl glycol diethylhexanoate, carbomer, parfum, aminomethyl propanol, limonene, hydroxycitronellal, citral, linalool

**G49**	Hospital (20/03/2022)	Antiseptic hydroalcoholic gel for healthy skin	Ethanol 70% w/w, phenoxyethanol 2.1% w/w, didecyldimethylammonium chloride 0.35% w/w, UNE-EN 1276, UNE-EN 1650, UNE-EN 14476, UNE-EN 12791, UNE-EN 13624, UNE-EN 14348, UNE-EN 13727
**G50**	Shop (19/03/2022)	Hydroalcoholic gel	Alcohol denat, aqua, isopropyl alcohol, glycerin, carbomer, sodium hydroxide.
**G51**	Shop (19/03/2022)	Hydroalcoholic gel	Alcohol denat, aqua, isopropyl alcohol, hydroxyethylcellulose, glycerin.
**G52**	Restaurant (19/03/2022)	Antiseptic hydroalcoholic gel for healthy skin	Ethanol 42%, propan-2-ol 22%, aqua, peg-15-cocamine, propylene glycol, glycerin, carbomer. UNE-EN 1040, UNE-EN 1500, UNE-EN 1276, UNE-EN 13624, UNE-EN 14476
**G53**	Personal use (19/03/2022)	Hydrating hand sanitizer	Alcohol denat, aqua, aloe barbadensis leaf juice, methyl gluceth-10, parfum, acrylates/C10-30 alkyl acrylate crosspolymer, alpha-isomethylionone, tromethamine, potassium sorbate, citronellol, limonene, linalool, hydroxycitronellol, sodium benzoate, eugenol.
**G54**	Personal use (19/03/2022)	Hydroalcoholic gel	Alcohol denat, aqua, glycerin, carbomer, allantoin, triethanolamine.
**G55**	Supermarket (25/03/2022)	Hands hydroalcoholic gel with aloe vera and glycerin	Alcohol Denat., Aqua, Glycerin, Propylene Glycol, Acrylates/ C10-30 Alkyl Acrylate Crosspolymer, Aloe Barbadensis Leaf Juice, Aminomethyl Propanol, Potassium Sorbate, Sodium Benzoate
**G56**	Laboral use (25/03/2022)	Hydroalcoholic gel. Hand sanitiser	Alcohol Denat, Aqua, Glycerin, **Phenoxyethanol**, Aloe Barbadensis Leaf Juice, Didecyldimonium Chloride.
**G57**	Restaurant (24/03/2022)	Hand cleaner gel	Alcohol Denat, aqua (water), hydroxypropyl methylcellulose, parfum (fragrance), glycerin, chlorhexidine digluconate, citric acid, sodium chloride, linalool, butylphenyl methylpropional, hexylcinnamal, citronellol, alpha-isomethyl ionone, eugenol, limonene
**G58**	Conference centre (24/03/2022)	Hydroalcoholic gel	Isopropanol, hydroxyethylcellulose, glycerin, water
**G59**	Bus (25/03/2022)	Hydroalcoholic	Alcohol denat, aqua, isopropyl alcohol, aloe barbadensis leaf extract, polyacrylate cosspolymer-6, parfum
**G60**	Restaurant (25/03/2022)	Sanitizing gel	Alcohol, aqua (water), isopropyl alcohol, glycerin, hydroxypropyl methylcellulose, menthol
**G61**	Restaurant (25/03/2022)	Antiseptic for healthy skin	71.5 % ethanol, glycerol, isopropyl, excipients c.s.p 100 %

**G62**	Restaurant (27/03/2022)	Hand sanitizer without water	Alcohol denat, aqua, hydroxyethylcellylose, phenoxyethanol, caprylyl glycol, farnesol, citric acid, sodium hydroxide
**G63**	Gambling hall (28/03/2022)	Hydroalcoholic gel	Alcohol denat, aqua, carbomer, aminomethyl lactate, propylene, glycol
**G64**	Personal use (24/03/2022)	Hydroalcoholic gel	Alcohol denat, aqua, hydroxyethylcellylose, triethanolamine
**G65**	Personal use (24/03/2022)	Hydroalcoholic gel with aloe vera	Alcohol denat, aqua, isopropyl alcohol, aloe barbadensis leaf juice, triethanolamine,carbomer, PEG-40 hydrogenated castor oil, trideceth-9, polysorbate-20, potassium sorbate, sodium benzoate, citric acid
**G66**	Personal use (25/03/2022)	Antiseptic lotion	Didecyldimethylammonium chloride 1%, n-propanol 40%, ethanol 14%, isopropanol 6,4%, excipients, aqua c.s.p. 100%
**G67**	Personal use (25/03/2022)	Sanitizing lotion	Alcohol denat, dimethyl ether, aqua, glycerin, aloe barbadensis leaf juice, potassium sorbate, sodium benzoate

Table S3. SPME-GC-MS/MS performance. Linearity, precision, recoveries, instrumental detection limits (IDLs) and limits of detection (LODs).

Compounds	Linearity		Precision. RSD (%)		Recovery ± Precision (%. RSD)	IDLs (ng L^{-1})	LODs (ng g^{-1})
	R^2	Linear range (µg L^{-1})	Intra-day (n=4)	Inter-day (n=5)			
Fragrance allergens							
Pinene	0.9942	0.05-5	4.5	13	91.3 ± 4.3	1.3	2.8
Limonene	0.9925	0.05-5	2.9	9.9	75.6 ± 4.3	2.2	2.8
Benzyl Alcohol	0.9922	0.05-5	5.1	13	78.0 ± 3.3	2.4	3.0
Linalool	0.9990	0.05-5	7.7	10	83.9 ± 1.5	1.0	1.3
Methyl-2-octynoate	0.9961	0.05-5	4.8	6.6	102 ± 2.3	17	27
Citronellol	0.9992	0.05-5	6.6	6.1	99.8 ± 9.3	1.9	3.0
Citral	0.9939	0.05-5	6.7	8.3	111 ± 3.3	3.7	4.0
Geraniol	0.9912	0.1-5	9.0	12	103 ± 5.7	1.9	4.3
Cinnamaldehyde	0.9996	0.1-5	11	12	108 ± 12	18	29
Anise alcohol	0.9912	0.1-5	8.0	14	112 ± 3.1	18	33
Cinnamyl alcohol	0.9909	0.1-5	1.1	7.9	108 ± 4.0	17	30
Eugenol	0.9906	0.05-5	8.5	10	116 ± 3.6	1.6	6.0
Methyleugenol	0.9993	0.05-5	12	11	106 ± 3.0	0.2	1.2
Isoeugenol	0.9920	0.05-5	5.8	9.9	103 ± 4.8	2.2	2.4
α-isomethylionone	0.9919	0.05-5	9.2	6.0	103 ± 2.9	1.5	2.6
Lilial®	0.9990	0.05-5	1.7	6.8	108 ± 2.4	0.16	2.7
Amylcinnamal	0.9949	0.05-5	12	14	116 ± 2.4	0.065	2.6
Amylcinnamyl alcohol	0.9942	0.05-5	6.3	16	107 ± 1.3	0.53	1.4
Farnesol	0.9964	0.05-5	8.7	9.7	112 ± 5.8	0.17	2.8
Hexylcinnamal	0.9964	0.05-5	9.7	12	111 ± 2.6	0.13	1.2
Benzyl benzoate	0.9959	0.05-5	12	9.1	81.5 ± 5.1	0.48	2.8
Benzyl salicylate	0.9989	0.05-5	10	16	96.7 ± 4.0	1.2	2.8
Benzyl cinnamate	0.9901	0.05-5	11	7.9	88.7 ± 4.2	2.3	2.6
Synthetic musks							
Musk Xylene	0.9931	0.05-5	7.8	10	110 ± 6.6	0.50	1.4
Musk Ambrette	0.9954	0.05-5	1.8	12	111 ± 3.2	0.010	1.1
Musk Moskene	0.9977	0.05-5	4.9	7.5	117 ± 2.8	0.026	3.3
Musk Tibetene	0.9926	0.05-5	3.0	8.6	106 ± 1.1	0.055	1.4
Galaxolide	0.9965	0.05-5	7.9	8.3	96.0 ± 3.9	0.78	2.0
Celestolide	0.9938	0.05-5	7.8	14	98.2 ± 1.8	0.049	1.0
Phantolide	0.9921	0.05-5	9.7	9.6	95.8 ± 1.4	0.76	3.2
Cashmeran	0.9959	0.05-5	6.8	16	102 ± 1.9	1.3	2.7
Traseolide	0.9935	0.05-5	9.7	9.9	95.3 ± 1.6	0.25	1.0
Tonalide	0.9945	0.05-5	11	7.9	95.4 ± 1.4	0.92	1.1
Ambrettolide	0.9957	0.05-5	3.2	5.9	96.2 ± 2.7	0.060	1.0
Preservatives							
PhEtOH	0.9980	0.1-5	7.9	4.6	102 ± 2.1	6.2	24
BHA	0.9989	0.05-5	5.2	5.8	106 ± 3.5	0.17	1.5
BHT	0.9950	0.05-5	7.3	7.0	104 ± 4.2	0.040	1.0
TCS	0.9940	0.05-5	9.2	13	103 ± 5.3	0.054	3.2
MeP	0.9964	0.05-5	5.7	8.0	102 ± 3.0	1.4	6.0
EtP	0.9943	0.1-5	13	11	102 ± 8.8	17	27
iPrP	0.9901	0.05-5	5.1	4.1	75.9 ± 4.2	4.3	13

PrP	0.9959	0.2-5	11	5.1	99.4 ± 7.7	15	65
iBuP	0.9965	0.1-5	12	16	96.0 ± 15	9.0	26
BzP	0.9939	0.05-5	2.7	11	97.3 ± 5.9	2.0	7.1
Plasticizers							
DMP	0.9923	0.02-5	12	11	106 ± 8.2	3.3	7.0
DEP	0.9943	0.1-5	5.7	13	n.c.	0.90	35
DIBP	0.9971	0.05-5	3.3	9.4	103 ± 4.6	0.039	7.1
DBP	0.9919	0.05-5	8.8	8.9	102 ± 4.2	0.33	2.5
DMEP	0.9940	0.05-5	11	14	94.9 ± 4.0	12	14
DIPP	0.9945	0.05-5	10	7.9	97.9 ± 5.2	1.9	3.3
DPP	0.9949	0.05-5	12	10	82.2 ± 6.7	0.51	3.5
BBP	0.9911	0.05-5	9.6	7.0	100 ± 6.9	0.85	4.1
DIHP	0.9982	0.05-5	4.7	8.2	107 ± 4.3	6.4	10
DEHP	0.9937	0.1-5	8.2	13	116 ± 2.5	3.4	26
DCHP	0.9980	0.05-5	8.2	5.8	95.4 ± 2.0	0.26	8.2
DPhP	0.9931	0.2-5	10	11	119 ± 5.2	0.71	34
DNOP	0.9919	0.05-5	6.9	13	107 ± 5.3	4.3	14
DMA	0.9922	0.05-5	6.8	11	81.6 ± 7.6	3.9	14
DEA	0.9929	0.05-5	6.4	8.3	90.9 ± 6.7	3.7	1.0
DEHA	0.9956	0.1-5	3.7	4.7	99.0 ± 10	0.39	20

Figure S1. Comparison of the recoveries obtained for fortified hydroalcoholic gel sample free of target compounds (except DEP) at two levels: 0.2 µg g^{-1} and 2 µg g^{-1}.

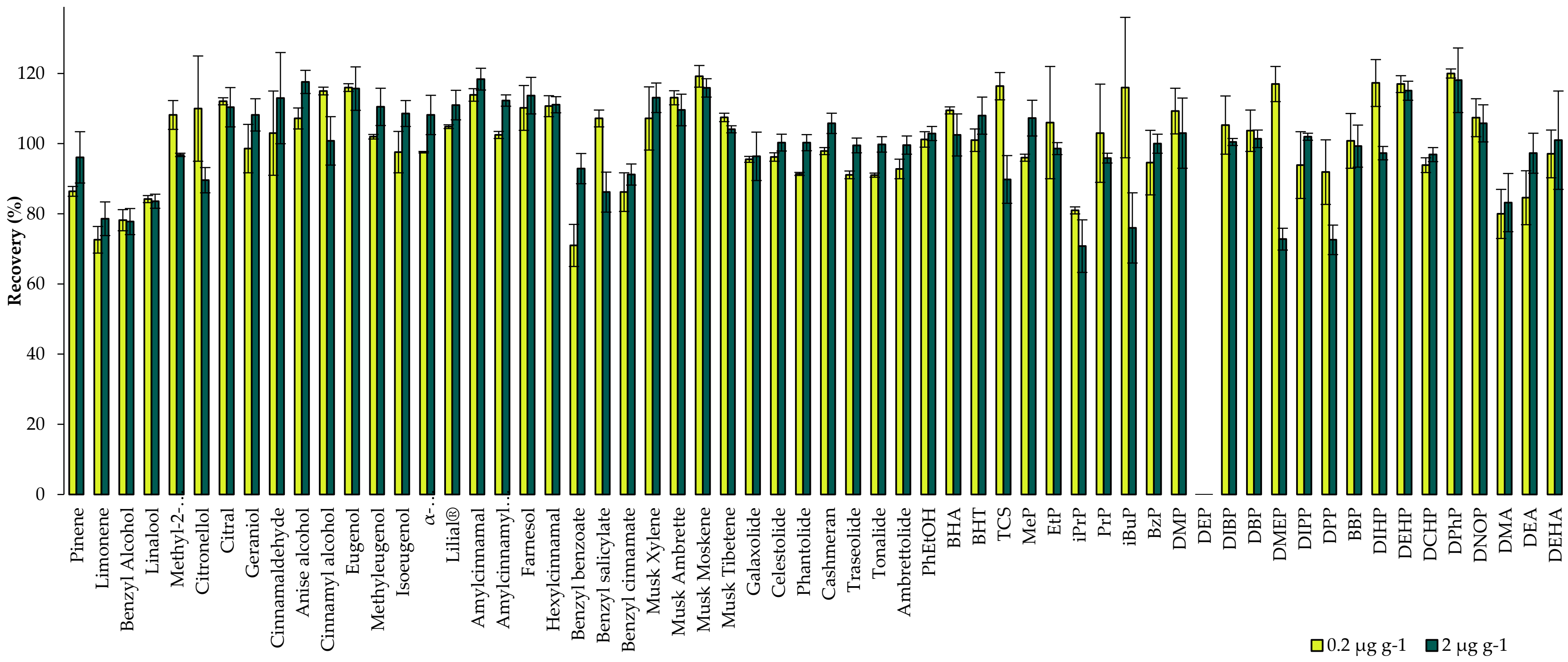

Supplement: Supplementary file 1 [file mps-06-00095-s001.zip › mps-2562433-supplementary.pdf]
